# Reconstitution of morphogen shuttling circuits

**DOI:** 10.1126/sciadv.adf9336

**Published:** 2023-07-12

**Authors:** Ronghui Zhu, Leah A. Santat, Joseph S. Markson, Nagarajan Nandagopal, Jan Gregrowicz, Michael B. Elowitz

**Affiliations:** ^1^ Howard Hughes Medical Institute and Division of Biology and Biological Engineering, California Institute of Technology, Pasadena, CA, USA.; ^2^ Department of Systems Biology, Harvard Medical School, Boston, MA, USA.

## Abstract

Developing tissues form spatial patterns by establishing concentration gradients of diffusible signaling proteins called morphogens. The bone morphogenetic protein (BMP) morphogen pathway uses a family of extracellular modulators to reshape signaling gradients by actively “shuttling” ligands to different locations. It has remained unclear what circuits are sufficient to enable shuttling, what other patterns they can generate, and whether shuttling is evolutionarily conserved. Here, using a synthetic, bottom-up approach, we compared the spatiotemporal dynamics of different extracellular circuits. Three proteins—Chordin, Twsg, and the BMP-1 protease—successfully displaced gradients by shuttling ligands away from the site of production. A mathematical model explained the different spatial dynamics of this and other circuits. Last, combining mammalian and *Drosophila* components in the same system suggests that shuttling is a conserved capability. Together, these results reveal principles through which extracellular circuits control the spatiotemporal dynamics of morphogen signaling.

## Teaser

Reconstitution of an extracellular protein circuit reveals how it actively “shuttles” signaling molecules away from their source.

## INTRODUCTION

In multicellular organisms, long-range signaling molecules called morphogens establish spatial concentration gradients to pattern developing tissues (*1*–*4*). The bone morphogenetic protein (BMP) signaling pathway is broadly conserved across metazoans and functions in nearly every tissue context. BMP ligands form gradients that control the patterning of early embryos (*5*, *6*), neural tube specification (*7*), and limb bud formation (*8*), among many other processes. BMP gradients need to be precisely controlled to ensure normal morphogenesis. For example, during early *Xenopus* embryo development, dorsal-ventral BMP gradients scale with embryo size to ensure a normal body plan, even when the embryo is cut in half (*9*–*11*). Similarly, in *Drosophila*, perturbations that affect the sharpness of orthologous decapentaplegic (Dpp) morphogen gradients alter developmental outcomes (*12*).

**Fig. 1.**
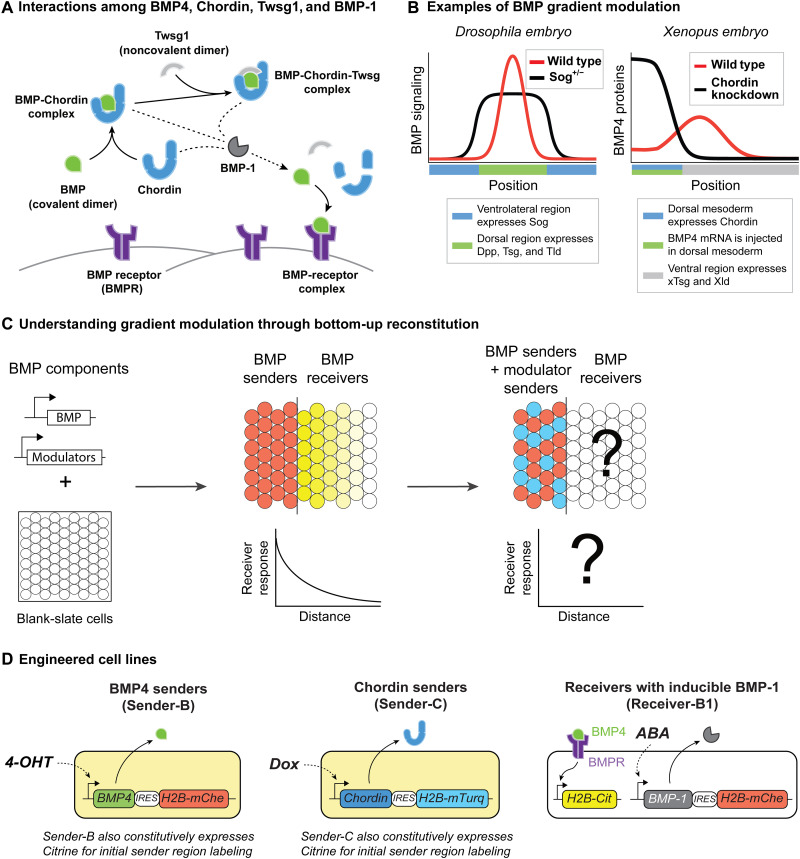
Synthetic gradient reconstitution enables systematic analysis of BMP gradient modulation. (**A**) Chordin can bind to BMP ligands, and Twsg1 strengthens this interaction. BMP-1 can cleave Chordin (dashed lines represent protein cleavage) in its free form or complex forms and release BMP for binding to receptor and signaling. (**B**) Schematic data. Left: In *Drosophila* early embryo, Dpp (*Drosophila* BMP ortholog), Tsg, and Tld are expressed dorsally. Ventrally expressed Sog (*Drosophila* Chordin ortholog) has been suggested to sharpen the BMP distribution to a narrower region. Tsg, *Drosophila* Twsg1 ortholog; Tld, *Drosophila* BMP-1 ortholog. Right: Chordin has been shown to push signaling away from the dorsal mesoderm after BMP4 injection into early *Xenopus* embryos (*10*). xTsg, *Xenopus* Twsg1 ortholog; Xld, *Xenopus* Twsg1 ortholog. (**C**) We reconstituted BMP gradients into “blank slate” NMuMG cells that express minimal levels of most morphogen pathway components aside from receptors and intracellular signal transducers (table S1) and exhibit minimal transcriptional changes in response to morphogen signaling (*41*–*43*). Synthetic pathway components were engineered into these cells to generate morphogen or modulator sender and receiver cells (left). Engineered senders and receivers were cocultured in different configurations (middle and right) to reconstitute BMP gradients. (**D**) We constructed BMP4 and Chordin senders and BMP4 receivers with inducible BMP-1 expression, such that circuit components BMP4, Chordin, and BMP-1 can be orthogonally controlled by 4-OHT, Dox, and ABA, respectively. Note that Sender-B and Sender-C also constitutively express Citrine for initial sender region labeling. mChe, mCherry; mTurq, mTurquoise2; Cit, Citrine. BMPR denotes BMP receptors, which are endogenously expressed in NMuMG cells (table S1). Details of these cell lines, and the constructs used to build them, are available in tables S2 and S3.

BMP gradients are shaped by a set of interacting extracellular components. Among them, the inhibitor Chordin, a cofactor termed Twisted gastrulation (Twsg1), and the BMP-1 protease (Fig. 1A) play key roles in shaping BMP ligand distributions (*13*, *14*). Chordin binds to BMP, preventing it from activating its receptors (*15*), and this interaction can be strengthened by Twsg1 (*16*–*19*). Thus, Chordin is mostly known as a BMP antagonist. However, Chordin has been postulated to actively redistribute BMP ligands across developing tissues, through a phenomenon termed “shuttling” (*20*, *21*). In this process, Chordin binds to BMP ligands, protecting them from receptor-mediated internalization and degradation (*22*). These Chordin-BMP complexes can diffuse extracellularly. Last, cleavage of Chordin by BMP-1 (*23*) can “release” BMP ligands at new sites. Depending on their expression domains, these components can generate shuttling processes that either displace signaling gradients away from the zone in which the ligand is produced (*10*, *24*–*29*) or compress signaling gradients into regions narrower than those in which the ligands are secreted (Fig. 1B) (*20*, *22*, *30*–*32*).

Despite much work characterizing the interactions among BMP ligands and modulators, key questions about how they work together as a system to shape signaling in space and time have remained unclear. First, are Chordin, Twsg, and BMP-1 sufficient for ligand shuttling? These components are all present during early zebrafish development. However, two recent studies argued against a role for shuttling (*33*, *34*). In this case, it is unclear whether other components (*26*, *27*) or feedback loops are required for shuttling, whether shuttling is actively suppressed, or whether the system is simply not operating in the appropriate parameter regime to enable shuttling. These possibilities are difficult to disentangle in the embryonic context, where one cannot readily isolate the effects of just a few components among the larger interconnected system. Second, what is the full repertoire of behaviors that a circuit consisting of Chordin, Twsg1, and BMP-1 can generate? While individual components have been perturbed in developing embryos, it has remained challenging to systematically and quantitatively tune multiple components in combinations. Third, is shuttling an evolutionarily conserved feature of the BMP pathway? Biochemical studies indicate that protein interactions among Chordin, Twsg1, and BMP-1 are largely conserved (*16*, *35*–*38*), but it remains unclear whether this leads to functional conservation of shuttling. Ideally, one would like to test conservation by combining components from different species, something that is difficult to do systematically in embryos.

An ideal way to address these questions is by reconstituting morphogen gradients outside of embryos (*39*, *40*). In this approach, engineered cells that produce circuit components or report on morphogen signaling can be arranged spatially in a cell culture system, allowing time-lapse imaging of spatiotemporal gradient dynamics. This bottom-up approach enables one to study pattern formation circuits in an isolated, quantitative manner and thereby test the sufficiency of specific component combinations for patterning behaviors, rationally engineer novel patterns, and analyze how evolutionary changes in circuit components affect patterning.

Here, we created a bottom-up gradient reconstitution system to study the BMP gradient formation process, analyze it in space and time, and perturb it through systematic, quantitative control of key modulators—Chordin, Twsg1, and BMP-1. We found that these components are sufficient to generate shuttling, resulting in displacement of gradients away from their ligand sources. They can also generate a repertoire of other spatiotemporal behaviors, including gradient lengthening and gradient suppression. Last, we found that *Drosophila* components can participate in shuttling circuits alongside their mammalian counterparts. The results can be integrated within a mathematical model that predicts how changes in component levels affect patterning. Together, these results establish shuttling as a conserved capability of the BMP system that emerges from a simple extracellular circuit of just a few interacting proteins.

## RESULTS

### BMP gradients can be reconstituted in confluent NMuMG monolayers

Reconstitution of BMP gradients requires sender cells that produce BMP morphogen and receiver cells that can respond to it. We used the NAMRU mouse mammary gland (NMuMG) epithelial cell line, which was previously shown to form confluent monolayers and to provide a high dynamic range of BMP signaling (*41*–*43*). We engineered a BMP4 sender cell line, Sender-B, that expresses BMP4 ligand together with a coexpressed mCherry reporter under the control of the inducer 4-hydroxytamoxifen (4-OHT) (Fig. 1D, left, and table S3) (*44*). We also constructed a Chordin sender cell line, Sender-C, that expresses Chordin under the control of a different inducer doxycycline (Dox) (Fig. 1D, middle; and table S3). For receiver cells, we used a previously described NMuMG cell line incorporating an Histone H2B (H2B)-Citrine reporter under the control of a BMP-responsive regulatory element, incorporating response elements from the BMP target gene Id1 (*43*). In this cell line, H2B-Citrine fluorescence is regulated by BMP in a dose-dependent fashion that correlates with the levels of phosphorylated Smad1/5/8 (pSmad) and with expression of endogenous BMP target genes (*42*, *43*) and can be detected within a few hours of BMP addition (fig. S1 and movies S1 to S4). We further integrated an abscisic acid (ABA)–inducible BMP-1 expression cassette (*45*) into this receiver cell line so that the resulting Receiver-B1 cell line can sense BMP4 as well as express BMP-1 under the control of ABA (Fig. 1D, right, and table S3). Last, we can control the level of Twsg1 by adding different concentrations of recombinant mouse Twsg1 protein (rTwsg1) to the culture. Together, these cell lines allow for the independent control of all circuit components in Fig. 1A.

**Fig. 2.**
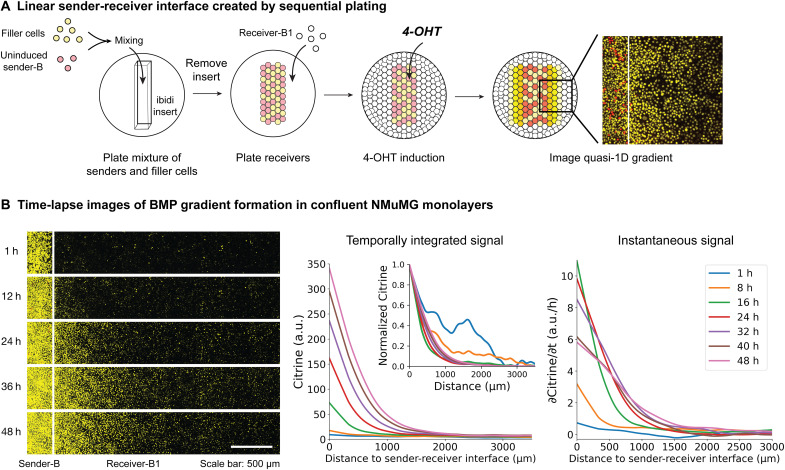
BMP gradients can be reconstituted in vitro. (**A**) To reconstitute gradient formation, we first used a PDMS insert to confine the plated senders in a confined region. Sender-B cells are mixed with filler cells that constitutively express Citrine. Then, we removed the insert and plated receivers in an unconfined region. After receiver plating, we induced the cells with 4-OHT in 50% Matrigel and imaged the BMP4 signaling gradient using fluorescence microscopy (see Materials and Methods). (**B**) Time-lapse imaging reveals gradient formation dynamics (also see movie S5). Left: In the sender region (left of the white line), Sender-C cells were used as the filler cells, and 4 μM 4-OHT was added to induce BMP4 expression in the sender cells. The mCherry fluorescence in the sender region can be more clearly seen in the individual channel images (fig. S2). Middle: Temporally integrated signal (measured by Citrine fluorescence) shows that the gradient shape approached a steady state at around 30 hours. This can be seen more clearly by the normalized Citrine signal (inset). The normalized Citrine signal for each time point was generated by (Citrine – Cit_min_)/(Cit_max_ – Cit_min_), where Cit_max_ and Cit_min_ are the maximum and minimum Citrine value for that time point, respectively. The 1- and 8-hour normalized curves are noisy due to low Citrine signals at early time points. Right: Instantaneous signal (measured by the time derivative of Citrine fluorescence) also peaked within 24 hours. Both temporally integrated signal and instantaneous signal are averaged over six replicates and smoothed (see Materials and Methods). In both (A) and (B), the white line on each image labels the position of the sender-receiver interface. The Citrine fluorescence is shown as yellow, and mCherry fluorescence is shown as red.

To reconstitute gradient formation, we used a sequential plating protocol similar to the one previously developed (*39*). Briefly, we first plated senders in a confined region using a polydimethylsiloxane (PDMS) insert. After overnight incubation, we removed the insert and plated receivers in an unconfined region. Twenty-four hours after receiver plating, we added a layer of 50% Matrigel on top of the cells to mimic an environment rich in extracellular matrix proteins. We added 4-OHT in the Matrigel to induce the secretion of BMP4 from the sender region and imaged the BMP4 signaling gradient using fluorescence microscopy (Fig. 2A). A quasi–one dimensional (1D) gradient of reporter fluorescence formed perpendicular to the interface between the senders and receivers (Fig. 2, fig. S2, and movie S5). By contrast, in the absence of 4-OHT induction of BMP expression, no gradient was observed (fig. S2B and movie S1). These results show that the spatially organized sender-receiver coculture can generate BMP signaling gradients.

To understand the spatiotemporal dynamics of gradient formation, we recorded time-lapse movies of the process. These movies revealed that BMP4 signaling amplitude increased over time as the gradient expanded in the length scale (Fig. 2B and movie S5). Gradient formation dynamics were consistent with a simple model of BMP4 gradient formation based on ligand production, diffusion, and removal (*46*). With very low Citrine degradation or dilution rate due to stability of H2B-Citrine and limited cell divisions, Citrine proteins accumulated from the start of the movie; thus, Citrine fluorescence reveals the temporally integrated output from the signaling reporter (see Materials and Methods). The normalized shape of this Citrine gradient approached a steady state at around 30 hours (Fig. 2B, middle inset). The time derivative of Citrine fluorescence is the instantaneous signal (Fig. 2B, right), which better reflects BMP concentrations (Materials and Methods). Instantaneous signals first increased near the source and then spread to more distal regions. After 30 hours, instantaneous signals began to diminish near the source, possibly due to receptor saturation, negative feedback by Smad6 or other regulators (*47*), or camera saturation at the highest intensity regions directly adjacent to the source. Together, these results show that BMP gradient formation can be reconstituted in a monolayer of engineered NMuMG cells and followed dynamically and quantitatively over time.

### The reconstituted system recapitulates interactions among BMP4 and its modulators

**Fig. 3.**
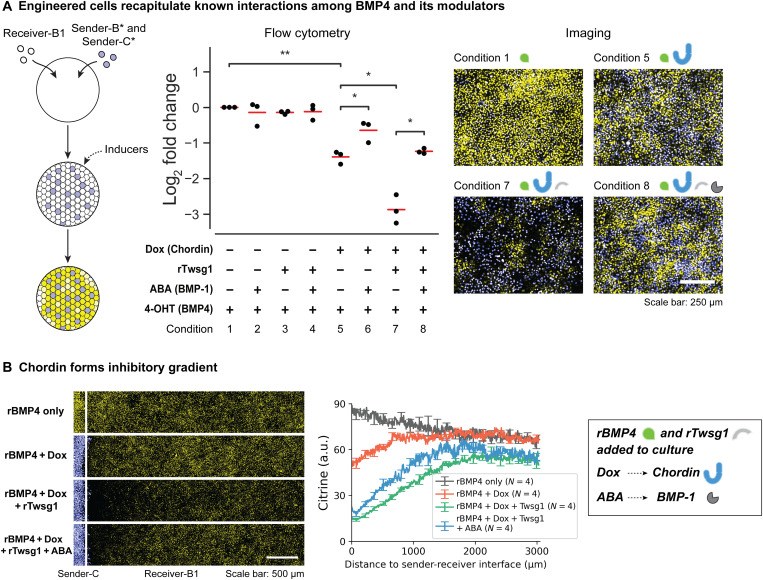
Engineered cells recapitulate known interactions among BMP4 and modulators. (**A**) Sender-receiver coculture experiments verify known interactions among circuit components. Left: Sender-B* cells and Sender-C* cells were engineered from Sender-B and Sender-C cells by adding constitutively expressed mTurquoise2 (table S3) so that they can be distinguished from Receiver-B1 cells. Middle: 4 μM 4-OHT was added to all samples. Each dot represents one biological replicate, and each red line indicates the mean of three replicates. For each replicate, log_2_ fold change was calculated by log_2_[(Receiver Citrine− Cit_0_)/(Cit_1_ − Cit_0_)], where Cit_0_ is the receiver Citrine of sample without 4-OHT (fig. S3), and Cit_1_ is the receiver Citrine of the sample with only 4-OHT induction (condition 1). *P* value based on Welch’s *t* test (fig. S3), *1 × 10^−2^ < *P* ≤ 5 × 10^−2^; **1 × 10^−3^ < *P* ≤ 1 × 10^−2^. Right: Images were taken right before flow cytometry measurements, which is 36 hours after induction. (**B**) The inhibitory gradients of Chordin can be modulated by Twsg1 and BMP-1. In samples with Dox induction, Dox was added 8 hours before other inducers to preinduce Chordin expression. In all samples, rBMP4 (15 ng/ml) was added to the culture, and we took images 24 hours after rBMP4 was added. *N* is the number of replicates. The white line on each image labels the position of sender-receiver interface. One of the replicates for rBMP4 + Dox + rTwsg1 + ABA used a smaller field of view due to an acquisition error, making the trace for that condition noisier (fig. S5). In both (A) and (B), Dox = 100 ng/ml, rTwsg1 = 10 nM, and ABA = 1000 μM. The Citrine fluorescence is shown as yellow, and mTurquoise2 fluorescence is shown as blue. White corresponds to yellow + blue on the computer screen. We removed the mCherry channel from images to avoid interfering with the Citrine visualization.

During development, extracellular modulators including Chordin, Twsg1, and BMP-1 interact with each other and with BMP4 ligand to regulate the spatial distribution of BMP signaling (Fig. 1A) (*13*, *48*). To verify that these components interact as expected, we mixed Sender-B (BMP4 senders), Sender-C (Chordin senders), and Receiver-B1 (BMP4 receivers with inducible BMP-1 expression) cells with different combinations of inducers (Fig. 3A, left), and analyzed reporter activation using flow cytometry (Fig. 3A, middle; and fig. S3) and fluorescence imaging (Fig. 3B, right). Inducing Chordin expression partially inhibited BMP4 signaling (Fig. 3A, condition 1 versus condition 5). The addition of recombinant Twsg1 (rTwsg1), which facilitates Chordin-BMP4 interactions (*16*), strengthened this inhibition, allowing Chordin to fully suppress BMP4 signaling (Fig. 3A, condition 7), but had minimal effect in the absence of Chordin (Fig. 3A, condition 3). Expression of the BMP-1 protease, which cleaves Chordin to release BMP4 ligands, partially relieved BMP4 inhibition by Chordin, both with or without Twsg1 (Fig. 3A, conditions 6 and 8). In a parallel experiment, we found that the inhibitor Noggin strongly suppressed signaling (fig. S4B). However, unlike Chordin, its effects were not reduced by BMP-1. All of these results are consistent with known interactions among BMP4, Chordin, Noggin, Twsg1, and BMP-1.

In vivo, Chordin and Noggin can diffuse away from their source to modulate BMP signaling in a spatially graded manner. Using the sequential plating protocol (Fig. 2A), we confined Sender-C cells to a central area of the plate, surrounded by Receiver-B1 cells. We induced localized Chordin production with Dox. Then, 8 hours later, we added recombinant BMP4 (rBMP4) to the whole culture. Localized Chordin production produced an inhibitory signaling gradient (Fig. 3B, row 2, and fig. S5). Further, addition of rTwsg1 enhanced the inhibitory gradient of Chordin (Fig. 3B, row 3, and fig. S5) while induction of BMP-1 reduced it (Fig. 3B, row 4, and fig. S5). A parallel experiment with Noggin senders also generated inhibitory gradients that could not be modulated by BMP-1 expression (fig. S4C). These results show that Chordin and Noggin can form diffusive inhibitory gradients.

### Mathematical modeling predicts gradient features

**Fig. 4.**
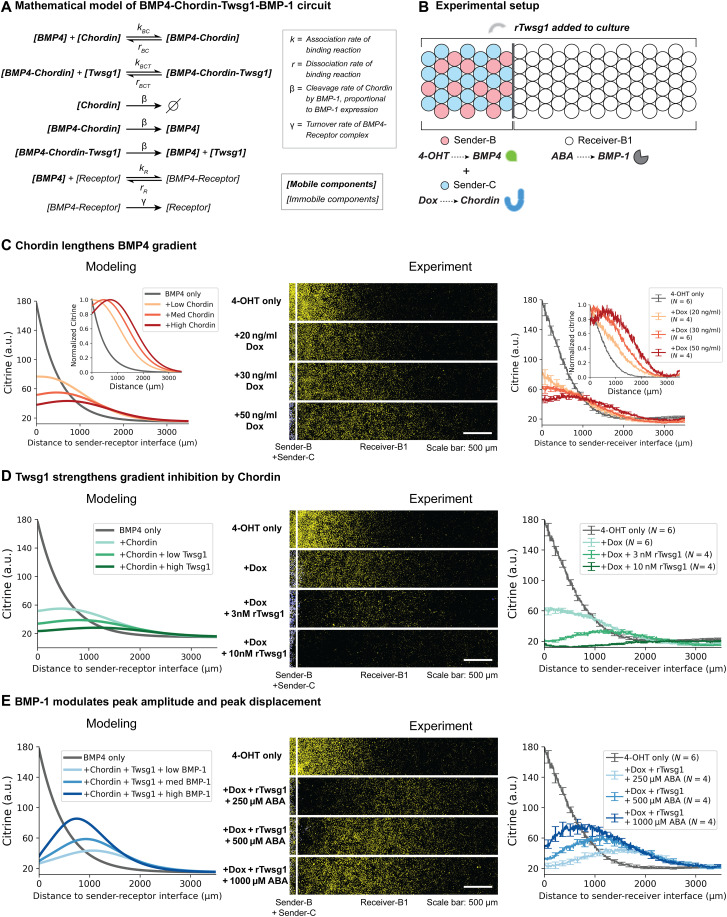
Mathematical modeling predicts and experiments confirm complex gradient modulation capabilities by Chordin, Twsg1, and BMP-1. (**A**) Mathematical model of BMP4, Chordin, Twsg1, and BMP-1 circuit. Model parameters were estimated based on existing literature (Supplementary Text and table S4). (**B**) The cells were plated in the same way as Fig. 2 (see Materials and Methods). 4-OHT, Dox, rTwsg1, and ABA were added at the same time and images were taken 48 hours after induction. (**C** to **E**) Tuning different circuit components enables diverse gradient modulation capabilities, including (C) gradient lengthening and amplitude dampening by tuning Chordin, (D) gradient suppression by tuning Twsg1, and (E) gradient displacement by tuning BMP-1. In (C) to (E), the white line on each image labels the position of sender-receiver interface. The Citrine fluorescence is shown as yellow, and mTurquoise2 fluorescence is shown as blue. White corresponds to yellow + blue on the computer screen. We removed the mCherry channel from images to avoid interfering with the Citrine visualization. 4-OHT (4 μM) was added to all samples. *N* denotes the number of replicates. In (D) and (E), Dox = 30 ng/ml. In (E), rTwsg1 = 10 nM.

To understand what types of patterns could be generated by these interactions, we developed a mathematical model of BMP and its modulators based on previous models (*10*, *20*, *30*) but restricted to the components analyzed here (Fig. 4A and Supplementary Text). In the model, BMP4 is secreted from sender cells, can be internalized through binding to receptors, undergoes diffusion, and forms complexes with Chordin. We further assumed that BMP4 and Chordin form a more stable complex in the presence of Twsg1 (*16*–*19*) and that BMP-1 cleaves Chordin both in its free form and complex forms at a rate proportional to BMP-1 expression. We used previous biochemical measurements of key parameters, including the BMP and Chordin diffusion coefficients; the binding and unbinding rates between BMP4, its receptors, and Chordin; and the rate of receptor-mediated internalization of BMP4 ligands (table S4). When parameter values were not available, we made arbitrary but physiologically reasonable assumptions (Supplementary Text and table S4) and later tested whether key conclusions were sensitive to these values (fig. S6). We further assumed that the rate of reporter expression follows a Hill function of the concentration of ligand-receptor signaling complexes. We then simulated the model in one spatial dimension corresponding to the quasi-1D gradients in the experiment.

Using the model, we first asked how each modulator affects the signaling gradient. In the simplest case in which BMP4 and Chordin are both produced in the sender cell region, Chordin both lengthened the gradient and reduced its amplitude, consistent with Chordin’s dual role of inhibiting and spatially extending BMP signaling (Fig. 4C, left) (*22*). In this case, BMP4 ligands could be released from the BMP4-Chordin complex due to their relatively weak affinity. In the presence of Twsg1, Chordin could completely abrogate the signaling gradient, because it binds to BMP4 more strongly (Fig. 4D, left).

Notably, introducing BMP-1 expression in simulated receiver cells generated a nonmonotonic displaced gradient. This represents ligand shuttling, as originally defined in the dorsal-ventral patterning of *Drosophila* early embryo (Fig. 1B, left) (*20*) but with a geometric arrangement more similar to that observed in the BMP4 injection experiment performed in the *Xenopus* early embryo (Fig. 1B, right) (*10*). Further, the level of BMP-1 controlled both the peak amplitude and peak displacement (distance of the peak position to the sender-receiver interface) of the shuttled signaling gradient. Higher BMP-1 expression elevated the peak amplitude and reduced peak displacement (Fig. 4E, left). Twsg1 was not strictly necessary for displacement of the gradient but strongly enhanced that displacement and increased the contrast between higher distal and lower proximal signaling (fig. S6C). Thus, mathematical modeling predicts that interactions among BMP4, Chordin, Twsg1, and BMP-1 enable diverse gradient modulation capabilities: Chordin alone reduces amplitude and extends the length scale, while Twsg1 with Chordin suppresses signaling, and BMP-1, in the presence of both Chordin and Twsg1, generates and modulates shuttling. These conclusions were relatively insensitive to the precise values of unknown parameters or to incorporation of a time delay to account for delays in Citrine expression and maturation (fig. S6).

### A minimal circuit consisting of BMP4, Chordin, Twsg1, and BMP-1 reconstitutes ligand shuttling

We next set out to experimentally determine whether shuttling could be reconstituted with these components and, more generally, test model predictions. We followed the same sequential plating protocol (Fig. 2A) to set up experimental gradients, using a mixture of Sender-B and Sender-C cells in the sender region, and Receiver-B1 cells in the receiver region (Fig. 4B). This configuration was inspired by the geometry of the BMP4 injection experiment performed in the *Xenopus* early embryo (Fig. 1B, right) (*10*).

Induction of Chordin production from the sender region lengthened the gradient while reducing its amplitude in a dose-dependent manner (Fig. 4C, right; fig. S7A), as predicted by the model (Fig. 4C, left). Normalized gradient further allows one to see this dose-dependent effect on gradient length independently of amplitude (Fig. 4C, left inset and right inset, fig. S7A). Consistent with coculture experiments (Fig. 3A) and model predictions (Fig. 4D, left), the global addition of rTwsg1 greatly enhanced the inhibitory effects of Chordin and completely suppressed BMP4 signaling gradients at a concentration of 10 nM (Fig. 4D, right; fig. S7B). Twsg1 alone minimally inhibited the gradients (fig. S8A). These results indicate that Chordin and Twsg1 largely behave as expected in the context of dynamic gradient formation.

Last, we analyzed the full shuttling circuit, including BMP4, Chordin, Twsg1, and BMP-1. Notably, when all components were present, the BMP4 signaling gradient was displaced from the sender region (Fig. 4E, right row 2, and fig. S7C). Crucially, the displaced gradient extended to a distance beyond that reached when only BMP4 was expressed (Fig. 4E, right row 2 versus row 1, and fig. S7C), indicating substantial ligand shuttling. Further, when the level of BMP4, Chordin, and rTwsg1 was fixed, modulating BMP-1 expression reduced peak displacement and elevated peak amplitude, consistent with model predictions (Fig. 4E, right, and fig. S7C). BMP-1 itself minimally affected gradients (fig. S8B) and cannot relieve Noggin inhibition (fig. S4, C and D). Thus, a minimal circuit with BMP4, Chordin, Twsg1, and BMP-1 enables generation and modulation of ligand shuttling.

### Dynamic properties of shuttling

From the developmental point of view, morphogen gradients allow cells to infer their positions within a spatially extended tissue. In principle, signaling could occur in a broad region and then subsequently sharpen to a narrower region or vice versa. It could also spatially shift over time toward more proximal or distal positions. These effects would affect how cells decipher morphogenetic information.

**Fig. 5.**
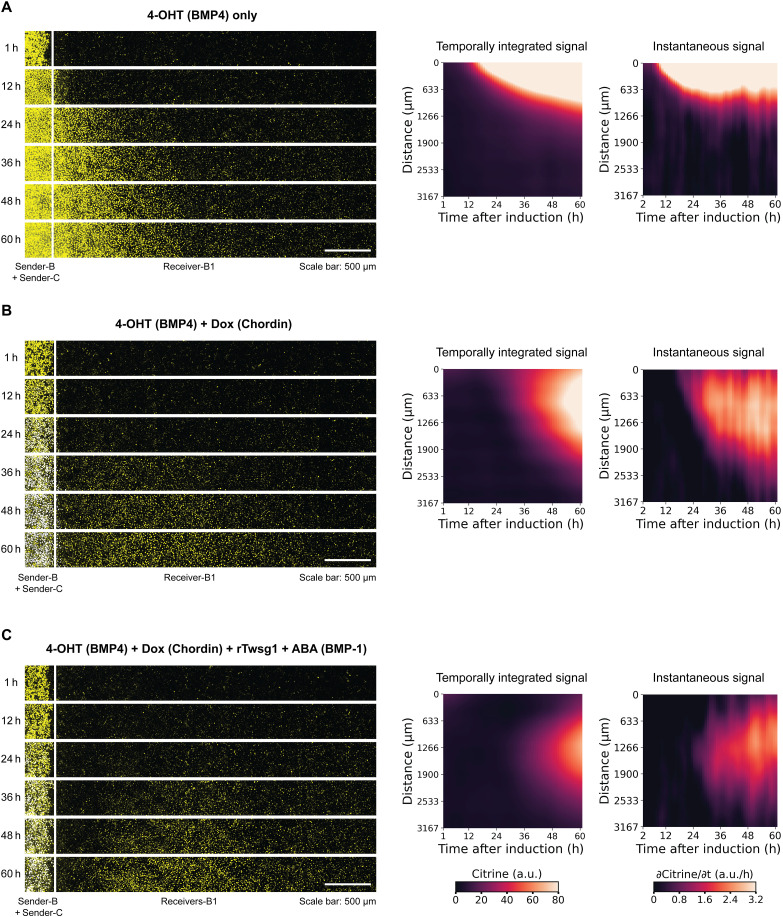
Time-lapse imaging reveals dynamic properties of shuttling. (**A** to **C**) Senders and receivers were plated in the same way as Fig. 4. The temporally integrated signal and instantaneous signal on the right are the average of six replicates and smoothed (see Materials and Methods). Left images are selected time points of movies S6 to S8 from one of the replicates. The white line on each image labels the position of sender-receiver interface. The Citrine fluorescence is shown as yellow, and mTurquoise2 fluorescence is shown as blue. White corresponds to yellow + blue on the computer screen. We removed the mCherry channel from images to avoid interfering with the Citrine visualization. 4-OHT = 4 μM, Dox = 30 ng/ml, rTwsg1 = 10 nM, and ABA = 500 μM.

To gain insight into gradient dynamics in different regimes, we recorded time-lapse movies of three selected regimes explored above: BMP4 only (Fig. 5A and movie S6), BMP4 with Chordin (Fig. 5B and movie S7), and the full circuit comprising BMP4, Chordin, rTwsg1, and BMP-1 (Fig. 5C and movie S8). With BMP4 alone, signaling initiates 6 hours after induction of BMP4 production (Fig. 5A, right). This delay exceeds that of the fluorescent reporter alone (fig. S1), likely due to additional delays between the addition of 4-OHT and secretion of BMP4 ligands. Once initiated, signaling first appeared at proximal regions and subsequently spread to more distal ones, reaching a saturating instantaneous signal gradient at ~30 hours (Fig. 5A, right). By contrast, with Chordin, it takes longer (not until ~30 hours) for instantaneous Citrine signals to reach a detectable level. Then, it spreads from proximal to distal regions (Fig. 5B). Last, in the full shuttling condition, with all components, instantaneous Citrine signals not only take longer to reach a detectable level, but also initiate near its final displaced peak position, and then spread outward from that position in both directions (Fig. 5C). Modeling results qualitatively recapitulate these dynamic features (fig. S9). This behavior could be adaptive in a developmental context because it prevents premature signaling away from the displaced signaling center that shuttling generates.

### Evolutionary conservation of shuttling

**Fig. 6.**
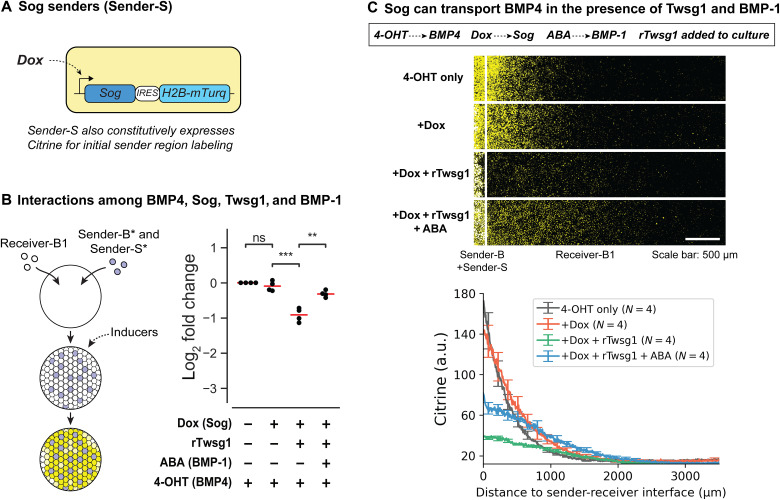
Sog transports BMP4 in the presence of Twsg1 and BMP-1. (**A**) We used the same Dox-inducible system as Sender-C to construct an inducible Sog sender cell line, Sender-S (table S3). (**B**) Sender-receiver coculture experiments show that inhibition of BMP4 signaling by Sog can be enhanced by Twsg1 and relieved by BMP-1. This coculture experiment was performed and quantified in a similar way to that in Fig. 3A, with Sender-S* substituting Sender-C*. Sender-S* cells were engineered from Sender-S cells by adding constitutively expressed mTurquoise2 (table S3) so that they can be distinguished from Receiver-B1 cells. Cellular fluorescence was measured 36 hours after induction by flow cytometry. Each dot represents one biological replicate, and each red line indicates the mean of four replicates. *P* value based on Welch’s *t* test (fig. S10), **1 × 10^−3^ < *P* ≤ 1 × 10^−2^; ***1 × 10^−4^ < *P* ≤ 1 × 10^−3^; ns: *P* > 0.05. (**C**) In the presence of Twsg1 and BMP-1, Sog can transport BMP4 to distal regions (row 4) exceeding that of BMP4 alone (row 1). Cells were plated using the Fig. 2A protocol, with Sender-S cells substituting filler cells. 4-OHT, Dox, rTwsg1, and ABA were added at the same time, and images were taken 48 hours after induction. The white line on each image labels the position of sender-receiver interface. The Citrine fluorescence is shown as yellow, and mTurquoise2 fluorescence is shown as blue. White corresponds to yellow + blue on the computer screen. We removed the mCherry channel from images to avoid interfering with the Citrine visualization. *N* denotes the number of replicates. In both (B) and (C), Dox = 500 ng/ml, rTwsg1 = 10 nM, and ABA = 250 μM.

The above results, together with evidence for shuttling in *Drosophila*, *Xenopus*, and mouse early development (*21*, *27*), suggest that shuttling is likely a conserved feature of the BMP pathway. To what extent have individual pathway components preserved biochemical features required for shuttling during evolution? The reconstituted system allowed us to create an evolutionary hybrid system by mixing components from different species. Taking advantage of this capability, we replaced Chordin with its *Drosophila* ortholog, Sog (28% sequence identity with mouse Chordin). We first created a Sender-S cell line that allows inducible expression of Sog, similar to the Sender-C line (Fig. 6A). We then repeated the coculture experiments as done in Fig. 3A, replacing Sender-C* cells with Sender-S* cells (Fig. 6B). Sog expression had minimal inhibitory effects on BMP4 signaling alone, possibly reflecting its substantial sequence divergence with mammalian Chordin. The inhibition was strengthened by rTwsg1 and was relieved by the further addition of BMP-1 (Fig. 6B and fig. S10). Thus, the key interactions of the mammalian circuit were preserved in the hybrid circuit, albeit with milder effects.

Using the sequential gradient protocol (Fig. 2A) to visualize the gradient in the hybrid circuit, we saw that the system also exhibited some similar features to its all-mammalian counterpart. We did not detect a statistically significant effect by Sog alone (Fig. 6C, row 2; and fig. S11). However, in the presence of rTwsg1, Sog significantly reduced signaling amplitude (Fig. 6C, row 3, fig. S11). The addition of BMP-1 did not displace the peak of the gradient (Fig. 6C, row 4, and fig. S11) as it did in the mammalian system (Fig. 4E). However, it did extend the gradient and produce a level of signaling at distal regions exceeding that of BMP4 alone (Fig. 6C, row 4 versus row 1, and fig. S11), consistent with distal transport of ligands. These results indicate that Sog is partially compatible with the mammalian circuit components and suggest that the interactions required for shuttling have been conserved during metazoan evolution.

## DISCUSSION

BMP, Chordin, Twsg1, and BMP-1 have been shown to enable ligand shuttling in *Drosophila* and *Xenopus* dorsal-ventral patterning (*10*, *20*, *30*). On the other hand, two recent studies in zebrafish embryos showed that BMP ligands were not shuttled despite the presence of all four circuit components (*33*, *34*), provoking the question of whether shuttling can occur in mammals and what minimal set of components is sufficient to generate it. By systematically reconstituting the shuttling circuit one component at a time, we were able to identify four distinct behaviors enabled by different combinations of components: BMP alone can form simple monotonically decreasing gradients (Fig. 2B); Chordin can delay and extend those gradients (Fig. 4C); Twsg1 with Chordin can suppress gradients (Fig. 4D); and BMP-1 with the other components can generate shuttling (Fig. 4E). In this last case, time-lapse movies revealed that gradients form at a distance from the source rather than propagating outward from it (Fig. 5).

A simple mathematical model shows how the four-component circuit is sufficient to enable ligand shuttling, notably predicts all of the four behaviors observed in these experiments, and highlights how the qualitative properties of the shuttling gradient depend on BMP-1 expression level. To do so, the model relies only on known interactions and previously measured parameters (table S4). Looking ahead, it will be important to identify more complex and developmentally relevant conditions in which the model fails, as additional components are included.

In natural developmental systems, the length scale of BMP gradients and the time scale of gradient formation vary markedly. Gradient lengths can be as small as a couple hundred micrometers in a *Drosophila* embryo (*49*), mouse vertebral field (*27*), or zebrafish pectoral fin (*50*), or as large as 1.5 to 2 mm in a *Xenopus* embryo (*51*). The time scale of gradient formation can be as fast as 30 to 60 min in a *Drosophila* embryo (*32*) or as long as several hours in a *Xenopus* (*52*) or zebrafish embryo (*50*, *53*, *54*). The length scale of gradient dynamics in our reconstituted system, 1 to 2 mm (Fig. 4), is similar to that in the *Xenopus* embryo, while the time scale (~24 hours) is significantly longer. A small part of this extended time scale can be explained by delays in reporter expression (fig. S1) and BMP induction (Fig. 5). However, even accounting for these delays, it appears that gradients develop faster in embryos than in the reconstituted system. An interesting challenge will be to see whether one can identify natural mechanisms that account for this acceleration and add them to the reconstitution. This will also require faster reporter systems that avoid the delays associated with fluorescence protein production and maturation and provide more instantaneous readout of pSmad levels.

Shuttling appears to be conserved during evolution. Biochemical and genetics studies have shown that interactions among BMP, Chordin, Twsg1, and BMP-1 are largely conserved across metazoans (*16*, *19*, *38*, *55*, *56*). These observations raise the provocative question of whether hybrid systems with components from distantly related organisms can still function (*57*, *58*). Our reconstituted system allows one to directly examine this question. By substituting *Drosophila* Sog for mouse Chordin, we found that some features of the resulting circuit are preserved, including gradient inhibition and lengthening. This substitution degraded the shuttling behavior, eliminating gradient displacement (Fig. 6C). This could be due to quantitative differences in BMP-Sog interactions, such as weaker binding. Another difference between *Drosophila* Sog and mammalian Chordin is that Tolloid cleavage of Sog is dependent on BMP binding (*12*, *59*), while BMP-1 can cleave vertebrate Chordin both in its free form and within a BMP-Chordin complex (*36*). While BMP-dependent cleavage is not necessary for shuttling, this mechanism has been suggested to increase the robustness of shuttling (*12*). It would be interesting to introduce *Drosophila* Tolloid into the reconstituted system and compare the robustness of shuttling enabled by *Drosophila* Sog and Tolloid, and mammalian Chordin and BMP-1. Analysis of other hybrid interspecies circuits across a broader range of expression levels could help to understand how evolutionary changes in molecular interactions cause changes in patterning.

These results show that shuttling can occur with mammalian components but do not imply that all BMP-Chordin dependent behaviors involve shuttling. The BMP-Chordin system appears capable of multiple qualitatively distinct patterning behaviors (*60*), depending on factors such as the expression level of circuit components or the expression configurations. For example, the configuration demonstrated here, where BMP and Chordin are expressed at the same region, resembles certain developmental contexts such as mouse vertebral field (*26*, *27*), and ectopic expression systems in embryos (*10*, *61*). However, it differs from the configuration in zebrafish early embryos, where BMP and Chordin are expressed from opposing poles, and a source-sink, rather than a shuttling, mechanism has been demonstrated (*33*, *34*). It is possible that the more linear gradient generated by the source-sink mechanism (*62*) is more desired in this developmental context. Future studies with the reconstituted system should allow investigation of other developmentally relevant configurations.

While we focused on shuttling, BMP pathway components generate a much broader range of behaviors, including scaling with embryo size (*10*, *11*) and spatially oscillatory patterning of complex tissues, such as digits (*8*). By extending the platform described here to incorporate additional components [e.g., other ligands (*42*, *43*), modulators (*13*), and receptors (*43*, *63*)], feedback loops in which signaling regulates pathway components, and other geometric configurations, one could, in principle, reconstitute other gradient behaviors and quantitatively explore these phenomena in a simplified setting. One could also analyze the role of co-occurring combinations of multiple BMP ligands such as BMP2, BMP4, and BMP7 in space and time (*64*, *65*). In addition, one could investigate the proposed ability of shuttling to enhance the robustness of gradient formation (*20*, *30*) and, within a larger expander-repressor system, to enable gradient scaling (*66*). By exploring a wide range of circuits, these experiments could also provide a foundation for the development of synthetic circuits to program multicellular pattern formation within the nascent field of synthetic developmental biology.

## MATERIALS AND METHODS

### Plasmid construction

Constructs used in this study are listed in table S2. Constructs were generated using standard cloning procedures. The inserts were generated using polymerase chain reaction or gBlock synthesis (Integrated DNA Technologies) and were annealed by Gibson Assembly with backbones that are linearized using restriction digestion. All of the construct maps are available at data.caltech.edu/records/0sdrn-73r13.

### Tissue culture

NAMRU mouse mammary gland cells (NMuMG, American Type Culture Collection, #CRL-1636) were cultured at 37°C in a humidity-controlled chamber with 5% CO_2_. The growth medium consisted of Dulbecco’s modified Eagle’s medium supplemented with 10% fetal bovine serum (VWR, #311 K18), penicillin (1 U/ml), streptomycin (1 μg/ml), 2 mM l-glutamine, and 1 mM sodium pyruvate. When trypsinizing cells for regular passage or for experiments, we first aspirated the medium and washed cells once with Dulbecco’s phosphate-buffered saline (PBS). After the wash, we aspirated the PBS and added 0.25% trypsin and incubated the cells at 37°C for 5 min and then added the medium to neutralize the trypsin.

### Recombinant proteins and chemicals

Recombinant mouse Twsg1 (R&D Systems, #756-TG) was dissolved in Dulbecco’s PBS with 0.1% bovine serum albumin at a stock concentration of 100 μg/ml. 4-OHT (Sigma-Aldrich #H7904) was dissolved in dimethyl sulfoxide (DMSO) at a stock concentration of 1 mM. Dox (Clontech Labs, #631311) was dissolved in Dulbecco’s PBS at a stock concentration of 100 μg/ml. ABA (Cayman Chemical, #10073) was dissolved in DMSO at a stock concentration of 100 mM.

### Cell line construction

Stable cell lines used in this study are listed in table S3. Stable cell lines were generated using the PiggyBac Transposon System (System Biosciences). Twenty-four hours before transfection, 100,000 NMuMG cells were seeded per well of a 24-well plate using a standard culture medium. The next day, cells were transfected with cotransfected transgene constructs in a PiggyBac expression backbone (table S2) and a Super PiggyBac Transposase plasmid using Lipofectamine LTX and PLUS Reagents (Thermo Fisher Scientific) according to the manufacturer’s protocol. Twenty-four hours after transfection, cells were transferred into a six-well plate and selected with corresponding antibiotics (350 μg/ml for hygromycin, 600 μg/ml for Geneticin, 400 μg/ml for Zeocin, and 10 μg/ml for blasticidin) for 6 days (split at day 3) to obtain a stable polyclonal population.

To obtain monoclonal population, the polyclonal population was diluted at 1 per well into 96-well plates. The plates were checked under a microscope after 4 to 5 days to eliminate wells without cells or with more than one colony. For wells that only have a single colony growing, cells were expanded, and subsequent screening (e.g., for inducibility) was performed to obtain monoclones. All the cell lines used in the current study are available from the corresponding author.

### Reconstitution of morphogen and modulator gradients in vitro by sequential plating

All gradient formation experiments were performed in regular 24-well tissue culture plates for gradients imaged at a fixed time point (24 hours for Fig. 3B and figs. S4C and S5 and 48 hours for Figs. 4 and 6C and figs. S4D and S8) or in a 24-well tissue culture–treated μ-Plate (ibidi, #82406) for time-lapse imaging (Figs. 2B and 5, fig. S2, and movies S1 to S8). To set up quasi-1D gradients, we first used tweezers to put PDMS culture insert (ibidi, #80209) in the well, such that one of the two rectangular spaces is at the middle of the well. Senders were trypsinized, counted, and plated in the center rectangular space. Specifically, we plated the following: 4000 Sender-B and 36,000 Sender-C for Figs. 2B, 3B, 4, and 5; figs. S2, S5, and S8; and movies S1 and S5 to S8; 4000 Sender-B and 36,000 Sender-N for figs. S4, C and D; and 5000 Sender-B and 65,000 Sender-S for Fig. 6C. After 24 hours, we used tweezers to remove PDMS culture insert and then trypsinized, counted, and plated 600,000 Receiver-B1 in the well. After 6 hours, we changed the medium to remove unattached cells. After 18 hours, we used two different induction protocols for morphogen gradient reconstitution and modulator gradient reconstitution. For reconstituting modulator gradients (Fig. 3B and figs. S4C and fig. S5), we changed the medium and added Dox (100 ng/ml) to the medium (except for the rBMP4 only wells) to preinduce Chordin or Noggin expression. After 8 hours, we aspirated the medium and added 250 μl of 50% Geltrex (Gibco, #A1413202, mixed with 50% medium) with rBMP4 (15 ng/ml) ± Dox (100 ng/ml) ± 10 nM rTwsg1 ± 1000 μM ABA. After 24 hours, we imaged the gradients. For reconstituting morphogen gradients (Fig. 2B, 4, 5, and 6C; figs. S2, S4D, and S8; and movies S1 to S8), we aspirated the medium and added 250 μl of 50% Matrigel (Corning, #CLS354230, mixed with 50% medium) with corresponding 4-OHT, Dox, rTwsg1, and ABA inducer combinations. We imaged the gradients either continuously using a time-lapse imaging platform (see below) or after 48 hours.

### Gradient imaging

For gradients imaged at a fixed time point after induction (24 hours for Fig. 3B and figs. S4C and S5 and 48 hours for Figs. 4 and 6C and figs. S4D and S8), we used EVOS Cell Imaging Systems (Thermo Fisher Scientific) with a 10X Olympus UPlanFL N objective [0.3 numerical aperture (NA)]. We used the scan mode of EVOS to acquire two rectangular areas at the left and right of the sender region (as two replicates), each region with an area of around 5000 μm × 4000 μm (usually 35 to 65 fields of view).

Time-lapse imaging of gradients or reporter dynamics (Figs. 2B and 5, fig. S2, and movies S1 to S8) was performed on an inverted Olympus IX81 fluorescence microscope with zero drift control, an ASI 2000YX automated stage, an iKon-M charge-coupled device camera (Andor), and a 20× dry objective (0.7 NA). Fluorescent proteins were excited with an X-Cite XLED1 light source (Lumen Dynamics). Microscope and image acquisition were controlled by Metamorph software (Molecular Devices). Cells were kept in a custom-made environmental chamber enclosing the microscope, controlling a humidified, 37°C and 5% CO_2_ atmosphere. For each well, two rectangular areas at the left and right of the sender region were imaged. Each region consists of 9 × 3 = 27 fields of view (each field of view is 650 μm × 650 μm, and we set a 50-μm overlap between adjacent two fields of view). Imaging started 1 hour after induction, and images were taken every 1 hour.

### Quantitative analysis of quasi-1D morphogen or modulator gradients

All image analysis was performed using customized Python scripts. For gradients imaged at a fixed time point after induction on EVOS (Figs. 3B, 4, and 6C, and figs. S4, C and D, S5, and S8), we stitched individual fields of view together to reconstruct images of the whole scanned region. During the stitching step, we also used images from medium-only wells and fluorophore-added wells to subtract medium autofluorescence and correct for uneven illumination. Specifically, fluorescence intensity at each pixel position was normalized by $FP=\frac{{FP}_{\mathrm{raw}}-{FP}_{\mathrm{back}}}{{FP}_{\mathrm{fluo}}-{FP}_{\mathrm{back}}}\times sc$, where *FP* is the normalized fluorescence intensity, *FP*_raw_ is the fluorescence intensity from raw data, *FP*_back_ is the fluorescence intensity from medium-only wells, *FP*_fluo_ is the fluorescence intensity of fluorophore-added wells, and *sc* is an arbitrarily chosen scaling factor.

The pixel-wise normalized fluorescence intensities of the scanned region were then loaded as a 2D matrix. The columns of the matrix were parallel to the sender-receiver interface. Next, we use the script to automatically detect sender-receiver interface through mTurquoise2 fluorescence channel (CFP channel) if Dox was added to the sample or through mCherry fluorescence channel (RFP channel) if Dox was not added to the sample. In either CFP channel or RFP channel, we first took the mean of fluorescence along the column. Then, we calculated a fluorescence difference profile by *FP*diff = *FP*mean(*i*) − *FP*mean (*i* + window), where *FP*mean (*i*) is the mean fluorescence level of the *i*th column, and we use a window = 300 pixels. If the sender region is at the right side of the image, the sender-receiver interface =argmin(*FP*diff). If the sender region is at the left side of the image, the sender-receiver interface =argmax(*FP*diff) + window. For samples with 4-OHT and ABA induction but not Dox induction (fig. S8B), the sender-receiver interface is manually determined. Last, the profiles of Citrine fluorescence channel (YFP channel) averaged along the column in the receiver region were plotted.

For time-lapse images of gradients or reporter dynamics (Fig. 2B and 5, fig. S2, and movies S5 to S8), we first stitched and normalized images similar to methods mentioned above. Because mCherry or mTurquoise2 fluorescence specific for sender regions was not turned on during the early time points of the imaging, we manually annotated the position of the sender-receiver interface. The Citrine fluorescence profiles averaged along the column in the receiver region were acquired at each time point and then were smoothed by a statsmodels.nonparametric.lowess function with a window of 0.15 along the time axis and distance axis.

The rate of Citrine fluorescence protein accumulation follows ∂Citrine/∂*t* = α − δ_cit_Citrine (Supplementary Text) in which α is the Citrine production rate and δ_cit_ is the Citrine removal rate. Because H2B-Citrine is very stable [with a half-life greater than 24 hours (*67*)] and cell proliferation is minimal during the imaging period, δ_cit_ is very small. Therefore, *∂Citrine/∂t* ≈ α ⇒ Citrine = ∫ α(*t*)*dt*. Citrine fluorescence thus is approximately the temporally integrated signal, and the time derivative of Citrine fluorescence *∂Citrine/∂t* is approximately the instantaneous signal. The instantaneous signal is an estimate of Citrine production rate α, which is a direct readout of BMP pathway signaling activity. To calculate the instantaneous signal, we first subtracted the smoothed Citrine profile of the current time point by the smoothed Citrine profile of the previous time point. Then, we subtracted the instantaneous signal of each sample by the instantaneous signal of the noninduced sample (fig. S2B and movie S1) to correct for background variations.

### Sender-receiver coculture experiments

The sender-receiver coculture experiments (Figs. 3A and 6B and figs. S3, S4B, and S10) were performed in regular 96-well tissue culture plates. Five thousand Sender-B*, 10,000 Sender-C*/Sender-N*/Sender-S*, and 50,000 Receiver-B1 were mixed and plated in the well. After 24 hours, we aspirated the medium and added 40 μl of 50% Cultrex (R&D Systems, #3432, mixed with 50% medium) with different inducer combinations. After 36 hours, Cells were trypsinized with TrypLE (Thermo Fisher Scientific, #12604013), spun down, and resuspended by flow cytometry buffer containing Hanks’ balanced salt solution (Life Technologies) and bovine serum albumin (2.5 mg/ml). Cell samples were then filtered by 40-μm cell strainers and analyzed by a flow cytometer (CytoFLEX, Beckman Coulter). We used the EasyFlow MATLAB-based software package developed by Y. Antebi to process flow cytometry data (https://antebilab.github.io/easyflow/). For each replicate, we also added a well of cells without any inducers. We calculate log_2_ fold change of Citrine by log_2_[(Receiver Citrine − Cit_0_)/(Cit_1_ − Cit_0_)], where Cit_0_ is the mean receiver Citrine of no induction sample and Cit_1_ is the mean receiver Citrine of sample with only 4-OHT induction.

## Supplementary Materials

### This PDF file includes:

Supplementary Text Figs. S1 to S11 Tables S1 to S4 Legends for movies S1 to S8 References

## Notes

View/request a protocol for this paper from *Bio-protocol*.
