# Supplementary material for: Reconstitution of morphogen shuttling circuits
Source: Sci Adv. 2023 Jul 12;9(28):eadf9336. doi: 10.1126/sciadv.adf9336 (PMC10337948; doi:10.1126/sciadv.adf9336)
Supplement: Supplementary file 1 — Supplementary Text Figs. S1 to S11 Tables S1 to S4 Legends for movies S1 to S8 References [file sciadv.adf9336_sm.pdf]

Science Advances
AAAS

Supplementary Materials for

Reconstitution of morphogen shuttling circuits

Ronghui Zhu *et al.*

Corresponding author: Michael B. Elowitz, melowitz@caltech.edu

Sci. Adv. **9**, eadf9336 (2023)
DOI: 10.1126/sciadv.adf9336

The PDF file includes:

Supplementary Text
Figs. S1 to S11
Tables S1 to S4
Legends for movies S1 to S8
References

Other Supplementary Material for this manuscript includes the following:

Movies S1 to S8

Supplementary Text

The mathematical model of BMP4-Chordin-Twsg1-BMP-1 circuit

Here we introduce the mathematical model of BMP4-Chordin-Twsg1-BMP-1 circuit, which is based on previous models (*10*, *20*, *30*) but specifically incorporating the components analyzed here (Fig. 4A), including mobile extracellular components BMP4 ($[BMP4]$), Chordin ($[Chordin]$), Twsg1 ($[Twsg1]$), BMP4-Chordin complex ($[BMP4\text{-}Chordin]$) and BMP4-Chordin-Twsg1 ($[BMP4\text{-}Chordin\text{-}Twsg1]$) complex, as well as immobile components receptors ($[R]$), BMP4-receptor complex ($[BR]$), and fluorescent reporters ($[Citrine]$).

We used a set of reaction-diffusion partial differential equations for mobile components. Thus, their equations have two parts. The first part is a diffusion term $D\nabla^2 c$, where c can be $[BMP4]$, $[Chordin]$, $[Twsg1]$, $[BMP4\text{-}Chordin]$, $[BMP4\text{-}Chordin\text{-}Twsg1]$, and D can be D_B, D_C, D_T, D_{BC}, D_{BCT}, correspondingly. We used the effective diffusion coefficients for each mobile component, taking into account the effects of their interactions with extracellular matrix components such as heparan sulfate polyglycans, but not BMP4-receptor interactions.

The second part contains reaction terms. First, BMP4 can bind to Chordin weakly, with estimated association rate k_{BC} and dissociation rate r_{BC}. Then BMP4-Chordin complex can further interact with Twsg1 to form BMP4-Chordin-Twsg1 complex, with association rate k_{BCT} and dissociation rate r_{BCT}. Furthermore, BMP4 can bind to receptors with estimated association rate k_R and dissociation rate r_R. Finally, Chordin in its free form or in complex forms can be cleaved by BMP-1 secreted by Receiver-B1 cells with a rate β dependent on BMP-1 expression level, and BMP4 and Twsg1 can be released from BMP4-Chordin and BMP4-Chordin-Twsg1 complex once Chordin is cleaved. Thus, we can write,

$$\begin{aligned}\partial[BMP4]/\partial t = {} & D_B\nabla^2[BMP4] - k_{BC}[BMP4][Chordin] + r_{BC}[BMP4\text{-}Chordin] \\ & - k_R[BMP4][R] + r_R[BR] + \beta[BMP4\text{-}Chordin] + \beta[BMP4\text{-}Chordin\text{-}Twsg1]\end{aligned}$$

$$\begin{aligned}\partial[Chordin]/\partial t = {} & D_C\nabla^2[Chordin] - k_{BC}[BMP4][Chordin] + r_{BC}[BMP4\text{-}Chordin] \\ & - \beta[Chordin]\end{aligned}$$

$$\begin{aligned}\partial[Twsg1]/\partial t = {} & D_T\nabla^2[Twsg1] - k_{BCT}[Twsg1][BMP4\text{-}Chordin] \\ & + r_{BCT}[BMP4\text{-}Chordin\text{-}Twsg1] + \beta[BMP4\text{-}Chordin\text{-}Twsg1]\end{aligned}$$

$$\begin{aligned}\partial[BMP4\text{-}Chordin]/\partial t = {} & D_{BC}\nabla^2[BMP4\text{-}Chordin] + k_{BC}[BMP4][Chordin] \\ & - r_{BC}[BMP4\text{-}Chordin] - k_{BCT}[Twsg1][BMP4\text{-}Chordin] \\ & + r_{BCT}[BMP4\text{-}Chordin\text{-}Twsg1] - \beta[BMP4\text{-}Chordin]\end{aligned}$$

$$\begin{aligned}\partial[BMP4\text{-}Chordin\text{-}Twsg1]/\partial t = {} & D_{BCT}\nabla^2[BMP4\text{-}Chordin\text{-}Twsg1] \\ & + k_{BCT}[Twsg1][BMP4\text{-}Chordin] - r_{BCT}[BMP4\text{-}Chordin\text{-}Twsg1] \\ & - \beta[BMP4\text{-}Chordin\text{-}Twsg1]\end{aligned}$$

Note that Twsg1 can also interact with BMP4 and Chordin individually (*71*), so there exist multiple interaction routes of forming the final BMP4-Chordin-Twsg1 complex. However, since interactions between Twsg1 and BMP4 or Chordin are much weaker than interactions between BMP4 and Chordin, we only consider one interaction route (BMP4 first interacts with Chordin, then BMP4-Chordin interacts with Twsg1) in our model.

For the immobile BMP4 receptors ($[R]$), other than reversible interactions with BMP4 ligands, we also considered receptor-mediated internalization and degradation of BMP4 ligands (*72*). Once the BMP4-receptor complex ($[BR]$) is internalized, we assumed that the BMP4 ligand is degraded and the receptor is recycled with a rate γ. Thus, we can write,

$$\partial[R]/\partial t = -k_R[BMP4][R] + r_R[BR] + \gamma[BR]$$

$$\partial[BR]/\partial t = k_R[BMP4][R] - r_R[BR] - \gamma[BR]$$

From these equations we can see that the total receptor concentration $RTotal = [R] + [BR]$ is held constant. Finally, we assume fluorescent reporter production rate follows a Hill function with BMP4-receptor complex concentration as a variable. The Citrine degrades with a ~24hr turnover time (*70*). Thus, we can write,

$$\partial[Citrine]/\partial t = b[BR]^n/(K^n + [BR]^n) - \delta_{cit}[Citrine]$$

To obtain more precise estimates of parameters that have relatively large estimated ranges from previous studies, such as $RTotal$, k_R, r_R and γ, as we as parameters that cannot be estimated from previous studies, such as b, n and K, we fitted a simplified model containing only $[BMP4]$, $[R]$, $[BR]$, $[Citrine]$, and without diffusion terms, to a time-lapse movie (Movie S2-4) of Receiver-B1 cells turning on Citrine fluorescence in response to 5, 10, 20 ng/ml recombinant BMP4. All estimated parameters used in the model are listed in Table S4.

There is a time delay for Citrine fluorescence to become detectable after BMP signaling, due to transcription, translation and maturation of Citrine fluorescence proteins (Fig. S1). To incorporate this delay into the model, we added a $[Citrine]^*$ term to lump together species like Citrine mRNA and immature Citrine proteins that are produced by Citrine fluorescence reporter but have not been converted into detectable mature Citrine proteins. The $[Citrine]^*$ can be converted into $[Citrine]$ with a conversion rate r_{cit}, i.e.,

$$\partial[Citrine]^*/\partial t = b[BR]^n/(K^n + [BR]^n) - \delta_{cit}[Citrine]^* - r_{cit}[Citrine]^*$$

$$\partial[Citrine]/\partial t = r_{cit}[Citrine]^* - \delta_{cit}[Citrine]$$

When parameter values were not available, we made arbitrary but physiologically reasonable assumptions, and later tested whether key conclusions were sensitive to these values. As shown in Fig. S6, key conclusions in this paper were relatively insensitive to the precise values of unknown parameters, or to the incorporation of time delay of Citrine fluorescence reporter.

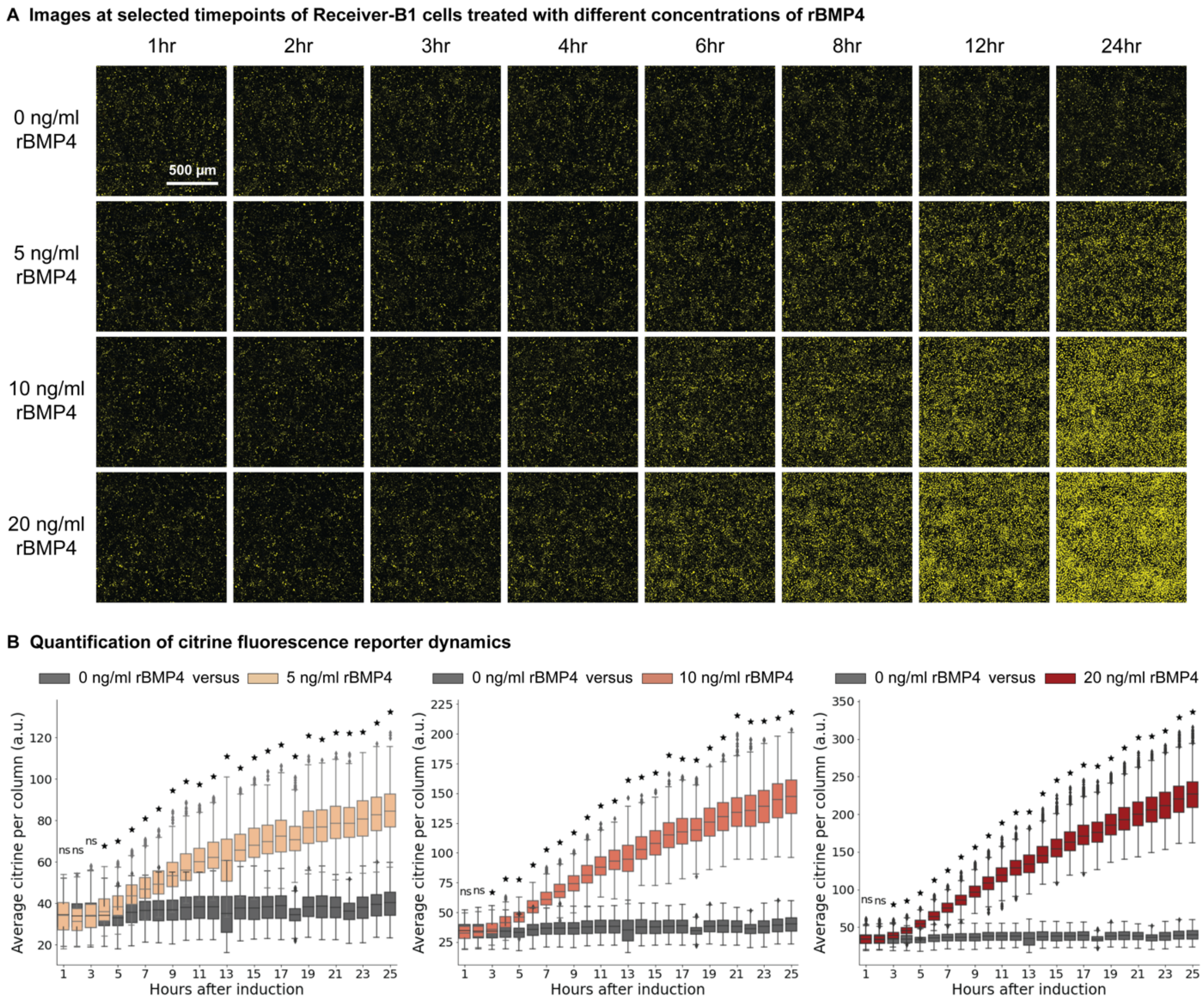

Fig. S1. Dynamics of citrine fluorescence reporter after recombinant BMP4 addition. (**A**) Images at selected timepoints of time-lapse imaging data (one of two replicates, corresponding to Movie S1-4) of Receiver-B1 cells treated with various concentrations of recombinant BMP4 (rBMP4). (**B**) Citrine fluorescence reporter shows significant elevation at 4 hours after the addition of 5 ng/ml rBMP4 (left), and at 3 hours after the addition of 10 ng/ml rBMP4 (middle) and 20 ng/ml rBMP4 (right). At each timepoint, we calculated the average citrine level per image column and acquired a distribution of average citrine per column. Then we used Welch's t-test to test whether citrine distribution of samples with rBMP4 is significantly larger than citrine distribution of the sample without rBMP4 for each timepoint (ns: $p \geq 0.05$, not significant; *: $p < 0.05$, significant). These results show that the time delay of Citrine fluorescence reporter is 3-4 hours, since BMP signaling can be detected by pSmad staining 20 minutes after rBMP4 addition (*44*).

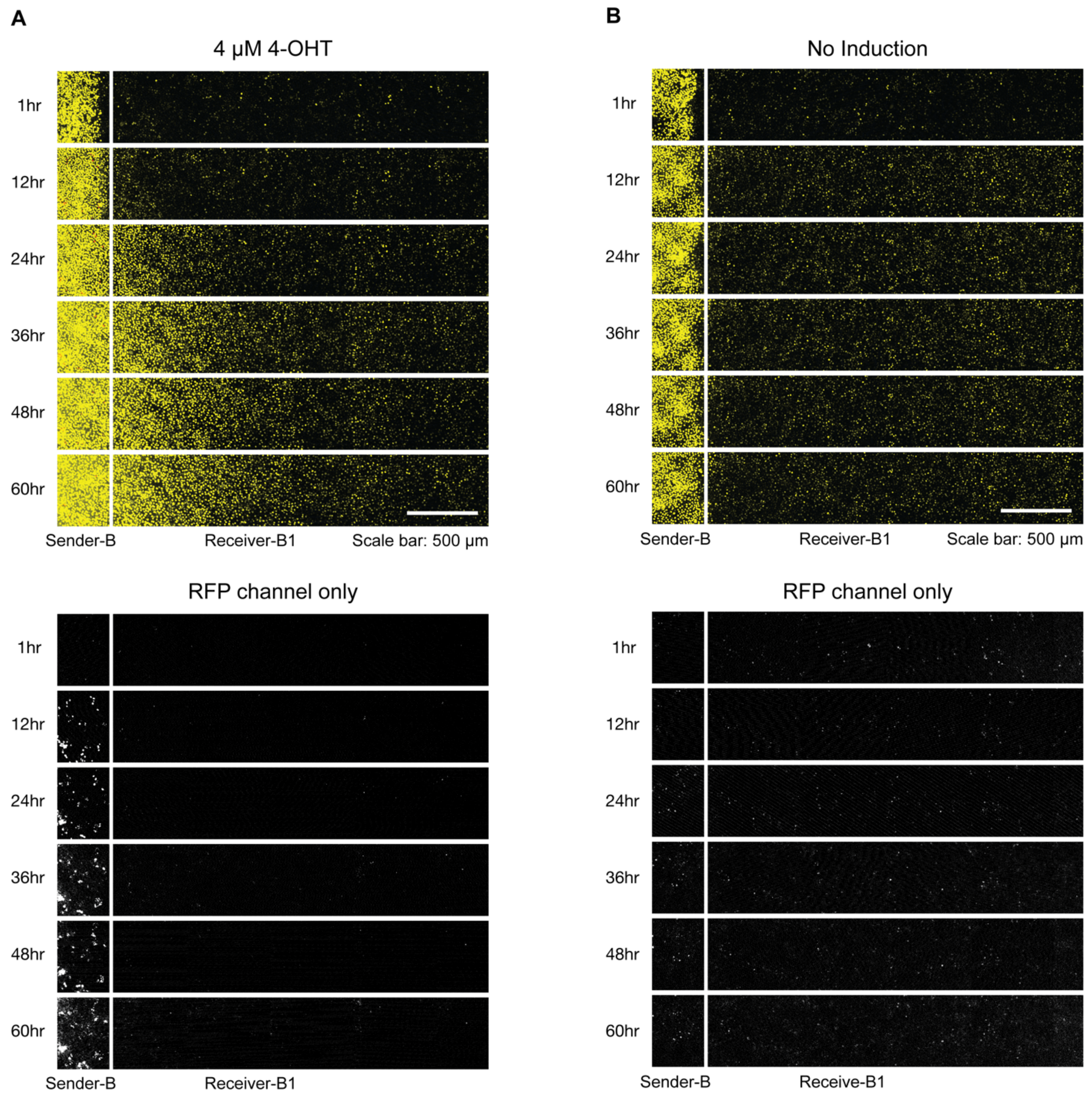

Fig. S2. BMP gradients can be reconstituted in vitro.
(**A**) (Top) Images at selected timepoints of Movie S5 are from the same time-lapse imaging data as Fig. 2B (adding a 60hr image). (Bottom) mCherry expression at the sender region can be visualized more clearly by focusing on the RFP channel alone at the same selected timepoints. (**B**) Without 4-OHT induction (Movie S1), no gradients were formed (top) and no mCherry expression (bottom) was detected in the sender region. In both (A) and (B), the white line on each image labels the position of sender-receiver interface. The Citrine fluorescence is shown as yellow, and mCherry fluorescence is shown as red.

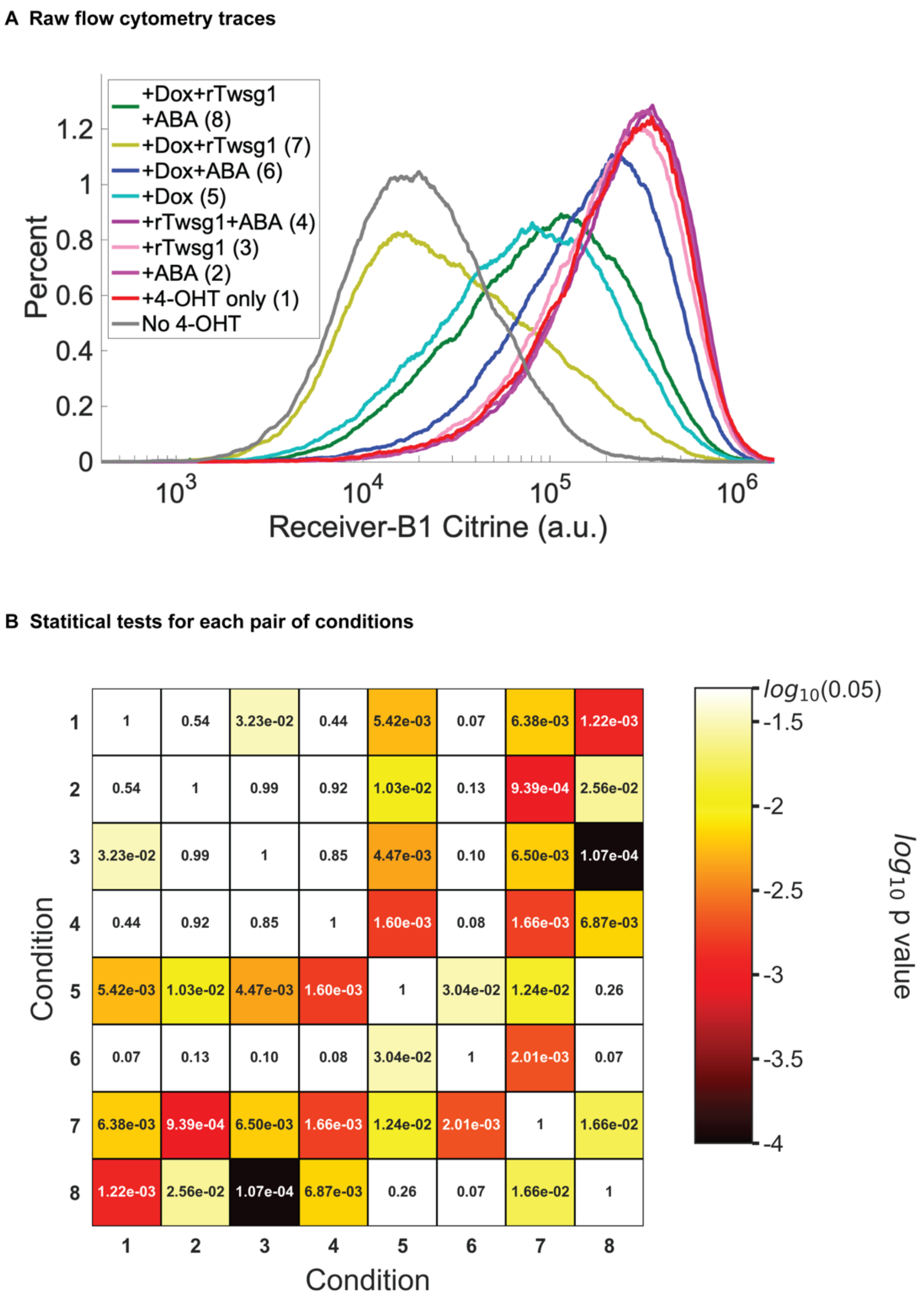

Fig. S3. Raw flow cytometry traces and statistical tests of sender-receiver co-culture experiment in Fig. 3A.

(**A**) These raw flow cytometry traces are from one of the three replicate experiments. To calculate the log2 fold change in Fig. 3A, we first obtained the mean Citrine from each trace, then calculated the log2 fold change by the equation $\log_2[(\text{Receiver Citrine} - Cit_0) / (Cit_1 - Cit_0)]$, where Cit_0 is the receiver Citrine of the sample without 4-OHT (gray trace) and Cit_1 is the receiver Citrine of the sample with only 4-OHT induction (red trace). The number in the parenthesis in the figure legend corresponds to the condition number in Fig. 3A. (**B**) For each matrix entry, the number is the p value of Welch's t-test between the corresponding pair of conditions. The condition number corresponds to the condition number in Fig. 3A.

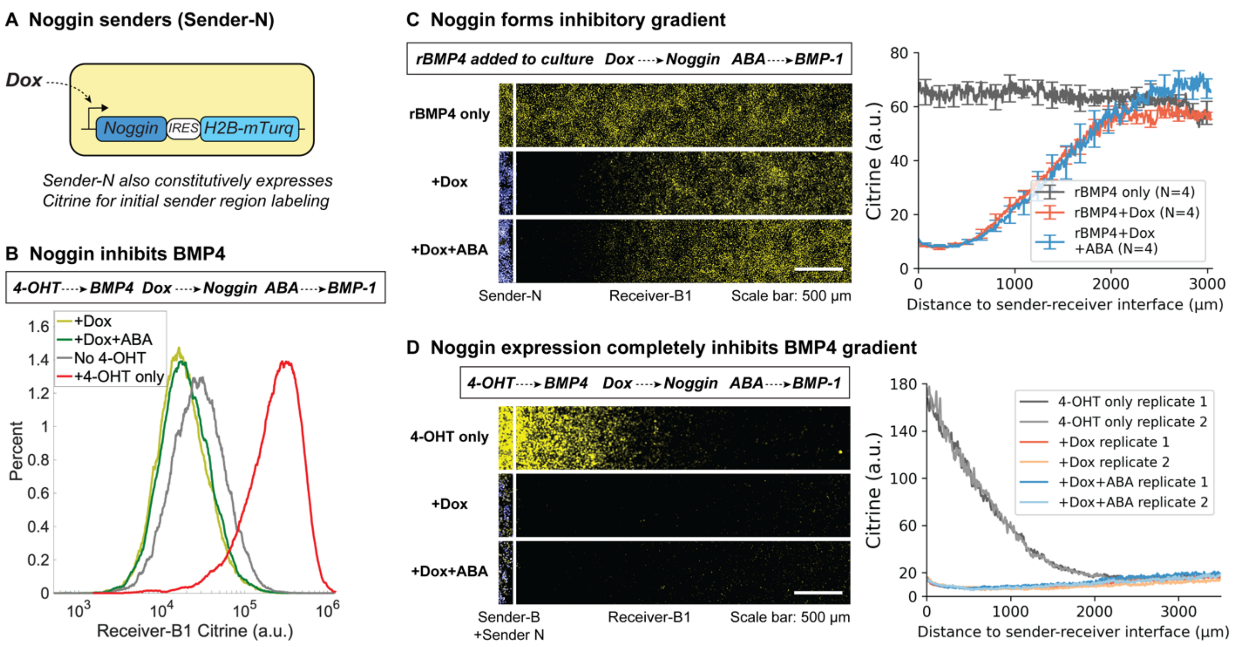

Fig. S4. Noggin strongly inhibits BMP4 signaling.

(**A**) We used the same doxycycline inducible system as Sender-C to construct an inducible Noggin sender cell line, Sender-N (Table S3). (**B**) Sender-receiver co-culture experiment verifies strong inhibition of BMP4 signaling by Noggin, which cannot be relieved by BMP-1 expression. This co-culture experiment was performed in a similar way to that in Fig. 3A, with Sender-N* substituting Sender-C*. Sender-N* cells were engineered from Sender-N cells by adding a constitutively expressed mTurquoise2 cassette (Table S3), so that they can be distinguished from Receiver-B1 cells in flow cytometry. (**C**) Noggin can form inhibitory gradients, which cannot be modulated by BMP-1 expression. In samples with Dox induction, Dox was added 8 hours before other components (rBMP4, rTwsg1 and ABA) to pre-induce Chordin expression. In all samples, 15 ng/ml recombinant BMP4 was added to the culture, and we took images 24 hours after rBMP4 was added. (**D**) Noggin expression completely abolishes BMP4 gradients. Cells were plated using the Fig. 2A protocol, with Sender-N cells substituting filler cells. 4-OHT, Dox and ABA were added together and images were taken 48 hours after induction. In both (B) and (D), 4-OHT = 4 μM. In (B), (C) and (D), Dox = 100 ng/ml, rTwsg1 = 10 nM, ABA = 1000 μM. In (C), N is the number of replicates. The white line on each image labels the position of sender-receiver interface. The Citrine fluorescence is shown as yellow, and mTurquoise2 fluorescence is shown as blue. White corresponds to yellow+blue on the computer screen. We removed the mCherry channel from images to avoid interfering with the Citrine visualization.

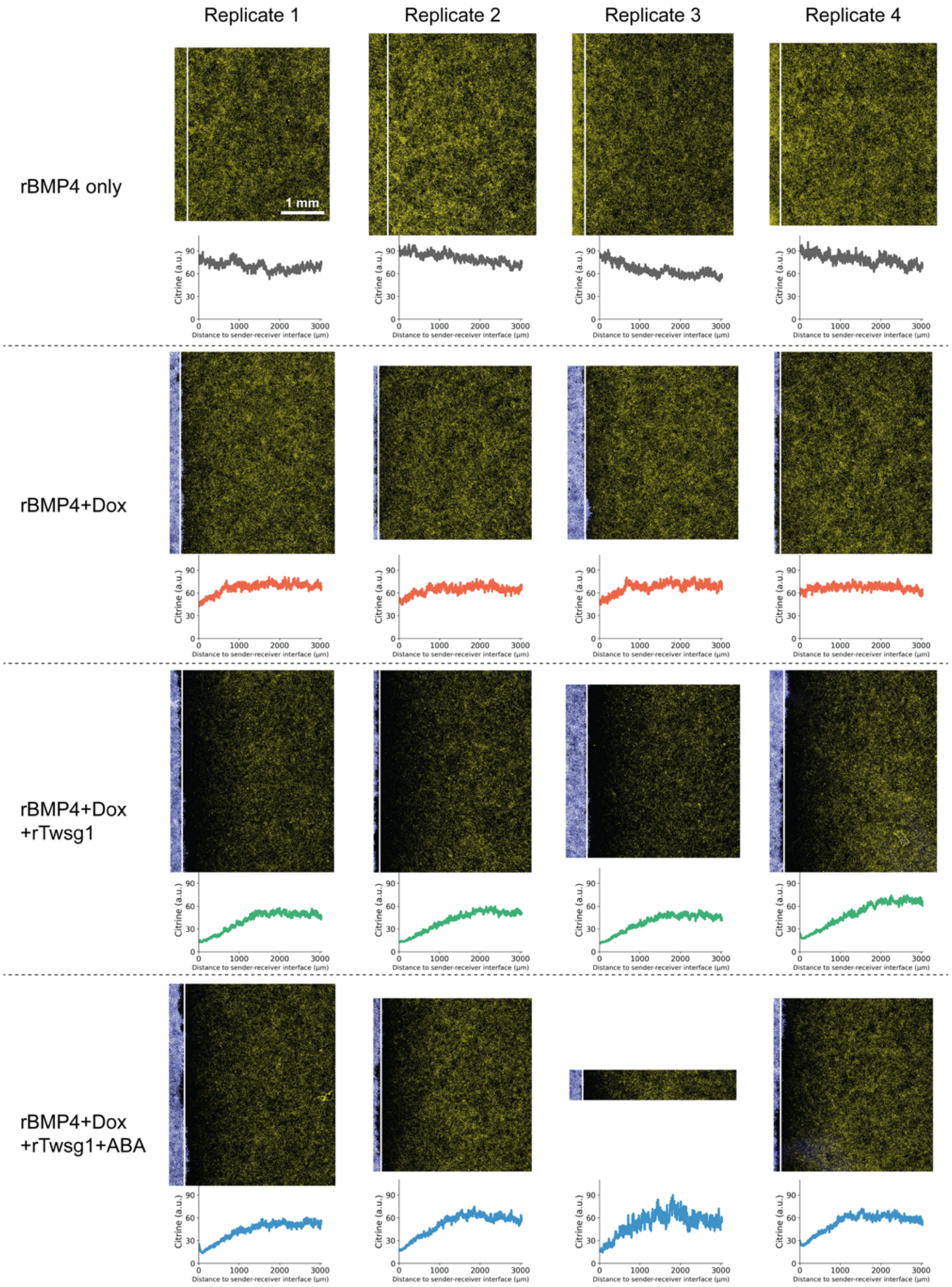

Fig. S5. Full images and individual traces of Fig. 3B.
The white line on each image labels the position of sender-receiver interface. The Citrine fluorescence is shown as yellow, and mTurquoise2 fluorescence is shown as blue. White corresponds to yellow+blue on the computer screen. We removed the mCherry channel from images to avoid interfering with the Citrine visualization. The replicate #3 of rBMP4+Dox+ rTwsg1+ABA group used a small field of view due to an acquisition error, making the individual trace and average trace for that condition noisier than other traces in Fig. 3B.

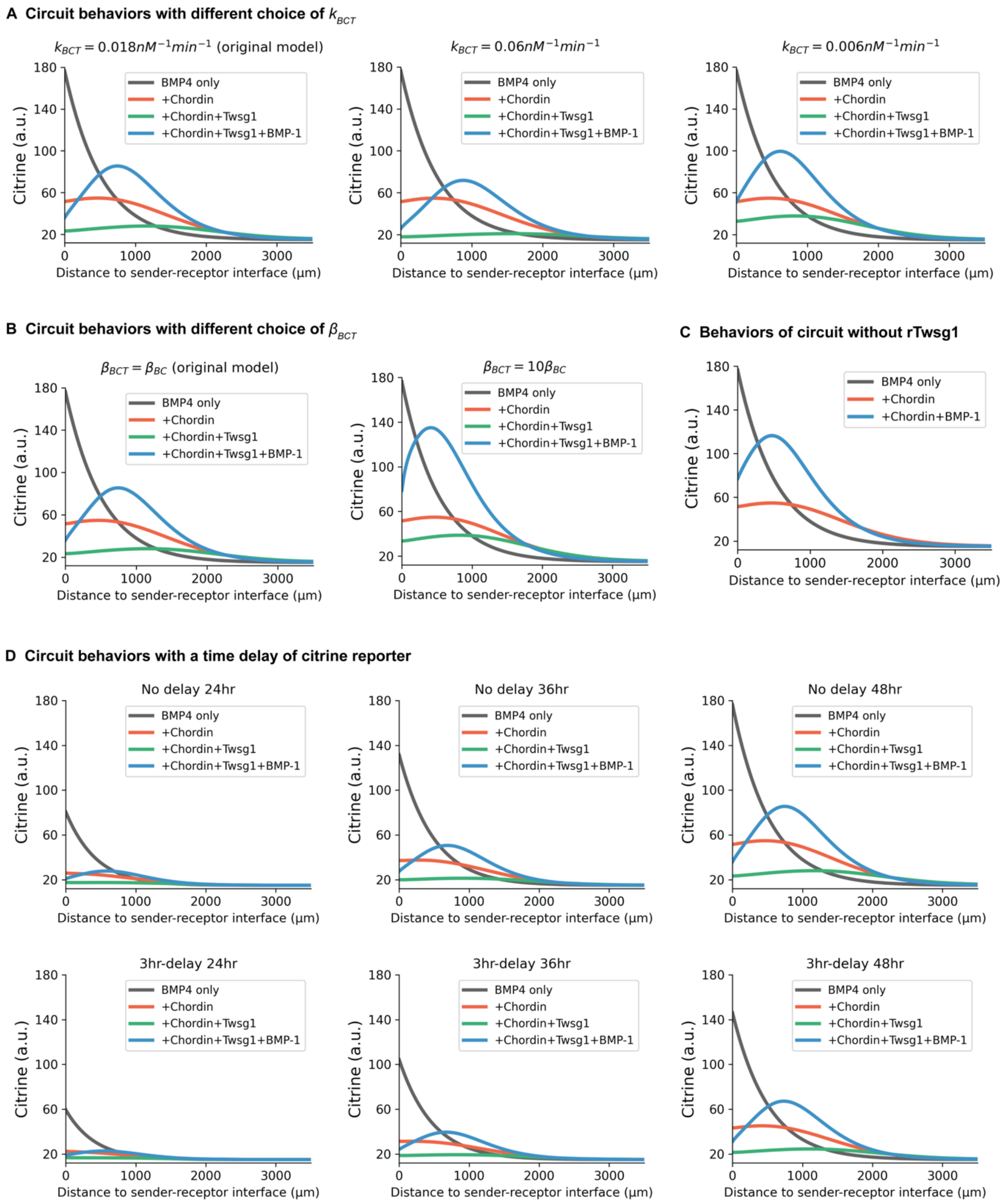

Fig. S6. Qualitative shuttling behaviors in Fig. 4 were insensitive to the precise values of unknown parameters or to incorporation of a time delay of Citrine reporter.

The qualitative shuttling behaviors include (1) Chordin lengthens the gradient, (2) Twsg1 with Chordin suppresses gradients, (3) BMP-1 with other components generates a displaced gradient. (**A**) In the original model (left), we arbitrarily chose a k_{BCT} to be the same as k_{BC} (Table S4), both within the diffusion limited rate range (0.006 $nM^{-1}min^{-1}$ – 0.06 $nM^{-1}min^{-1}$) (*73*). Holding other parameters constant, changing k_{BCT} to the upper (middle) or lower (right) limit of the diffusion

limited rate range does not affect the qualitative shuttling behaviors. (**B**) Previous studies showed that Twsg1 enhances BMP-1 cleavage of Chordin *in vitro* (*18, 74*). In the original model (left), we set the BMP-1 cleavage rate β to be the same for Chordin in both BMP4-Chordin and BMP4-Chordin-Twsg1. Holding other parameters constant, setting a higher BMP-1 cleavage rate for Chordin in BMP4-Chordin-Twsg1 (right) does not affect the qualitative shuttling behaviors. (**C**) Twsg1 is not necessary for a displaced gradient, but the contrast between proximal and distal BMP signals (compared with the plots of the original model in (A) or (B)). (**D**) Incorporating the time delay of Citrine reporter (Fig. S1) does not affect the qualitative shuttling behaviors. We incorporate the time delay by introducing a $[Citrine]^*$ term to lump together species that are produced by Citrine fluorescence reporter but not detectable yet, such as Citrine mRNA and immature Citrine fluorescence proteins, and a conversion rate r_{cit} between $[Citrine]^*$ and detectable mature Citrine fluorescence protein $[Citrine]$ (Supplementary Text). The r_{cit} is set to 0.00385 min^{-1}, corresponding to a 3-hour conversion time (Table S4, Fig. S1).

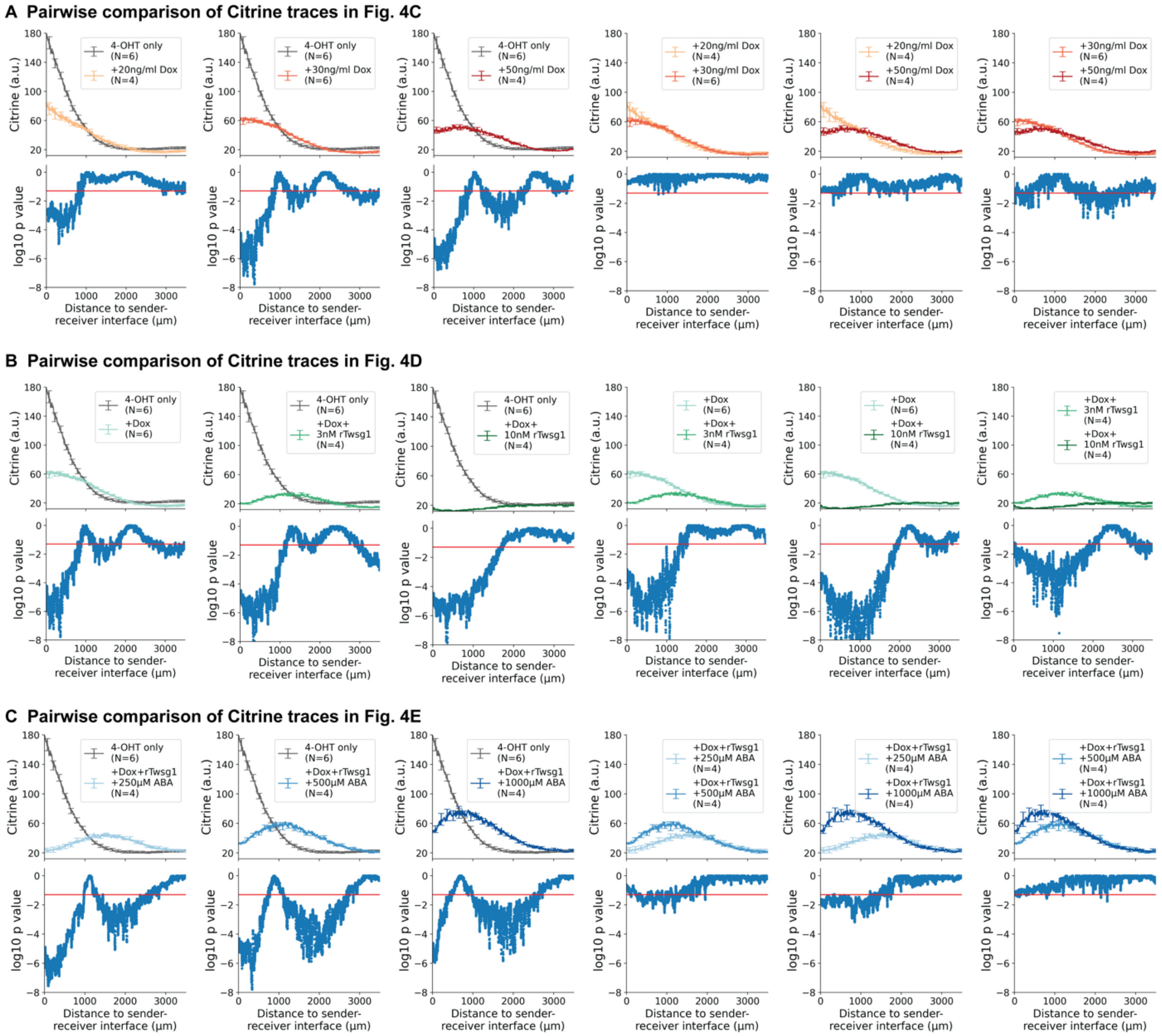

Fig. S7. Pairwise comparison of Citrine traces in Fig. 4.

For each pair of traces, we performed pixel-wise Welch's t-test along the distance to the sender-receiver interface. The red line on the distance versus log10 p value plot is $\log_{10}(0.05)$.

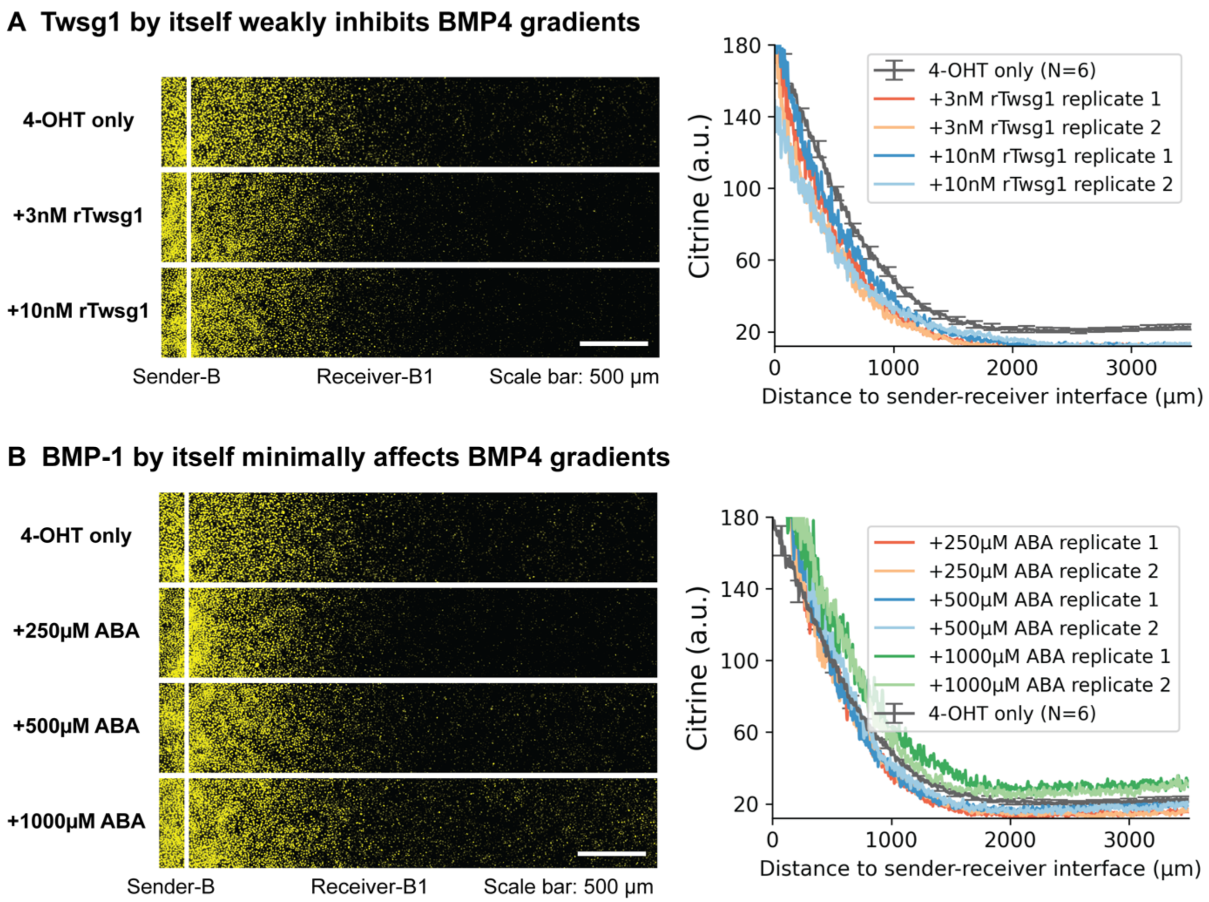

Fig. S8. Twsg1 addition or BMP-1 expression minimally affects BMP4 gradients without Chordin.

In the sender region, Sender-B cells were mixed with filler cells (Sender-C, see Materials and Methods). 4-OHT and (A) rTwsg1 or (B) ABA were added together, and images were taken 48 hours after induction. In both (A) and (B), the white line on each image labels the position of sender-receiver interface. The Citrine fluorescence is shown as yellow. We removed the mCherry channel from images to avoid interfering with the Citrine visualization. 4 μM 4-OHT was added to all samples. N denotes the number of replicates.

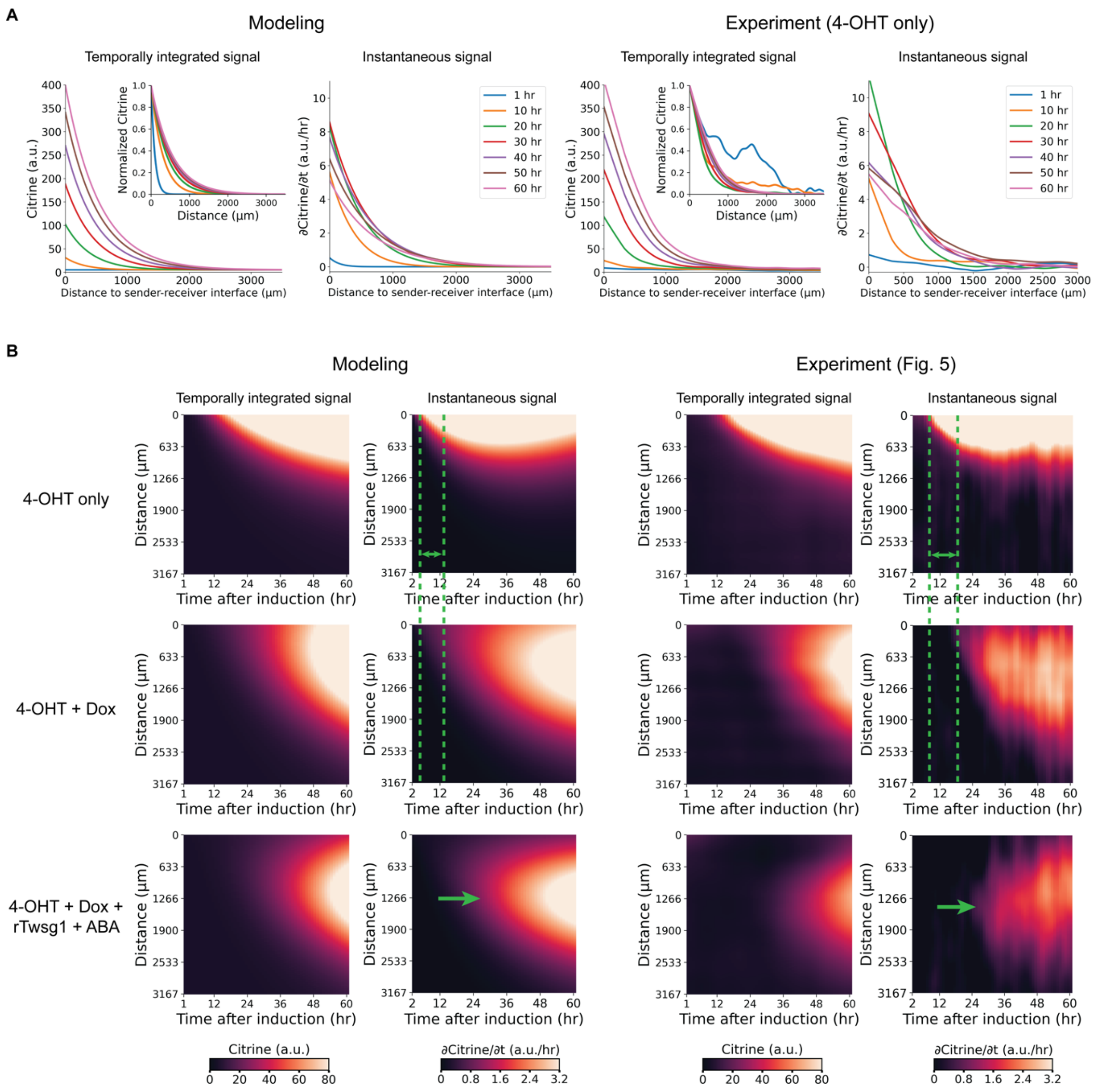

Fig. S9. The mathematical model recapitulates the dynamic properties of shuttling.
We generated the simulated dynamic gradient formation data using the same mathematical model in Fig. 4 (also see Supplementary Text), with all the parameters being the same except for the scaling factor of Citrine fluorescence (*b*). (**A**) Simulated data recapitulates the dynamic features of gradient formation for 4-OHT only condition (Fig. 2B and Fig. S2A), including gradient shapes approach a steady state at around 30hr (red line of normalized citrine) and instantaneous signal first increases near the source, then spread to more distal regions, and begin to diminish near the source after 30hr, possibly due to receptor saturation. (**B**) Simulated data recapitulates the dynamic features of shuttling. The dotted lines and bidirectional arrows between dotted lines in the top two instantaneous signal plots show that, both in model and in experiment, Chordin addition by Dox causes a delay of the Citrine signal to reach a detectable level. The arrows in the bottom instantaneous signal plot show that, both in model and in experiment, the Citrine signal in 4-OHT+Dox+rTwsg1+ABA condition initiates near the final displaced peak position and spreads outwards.

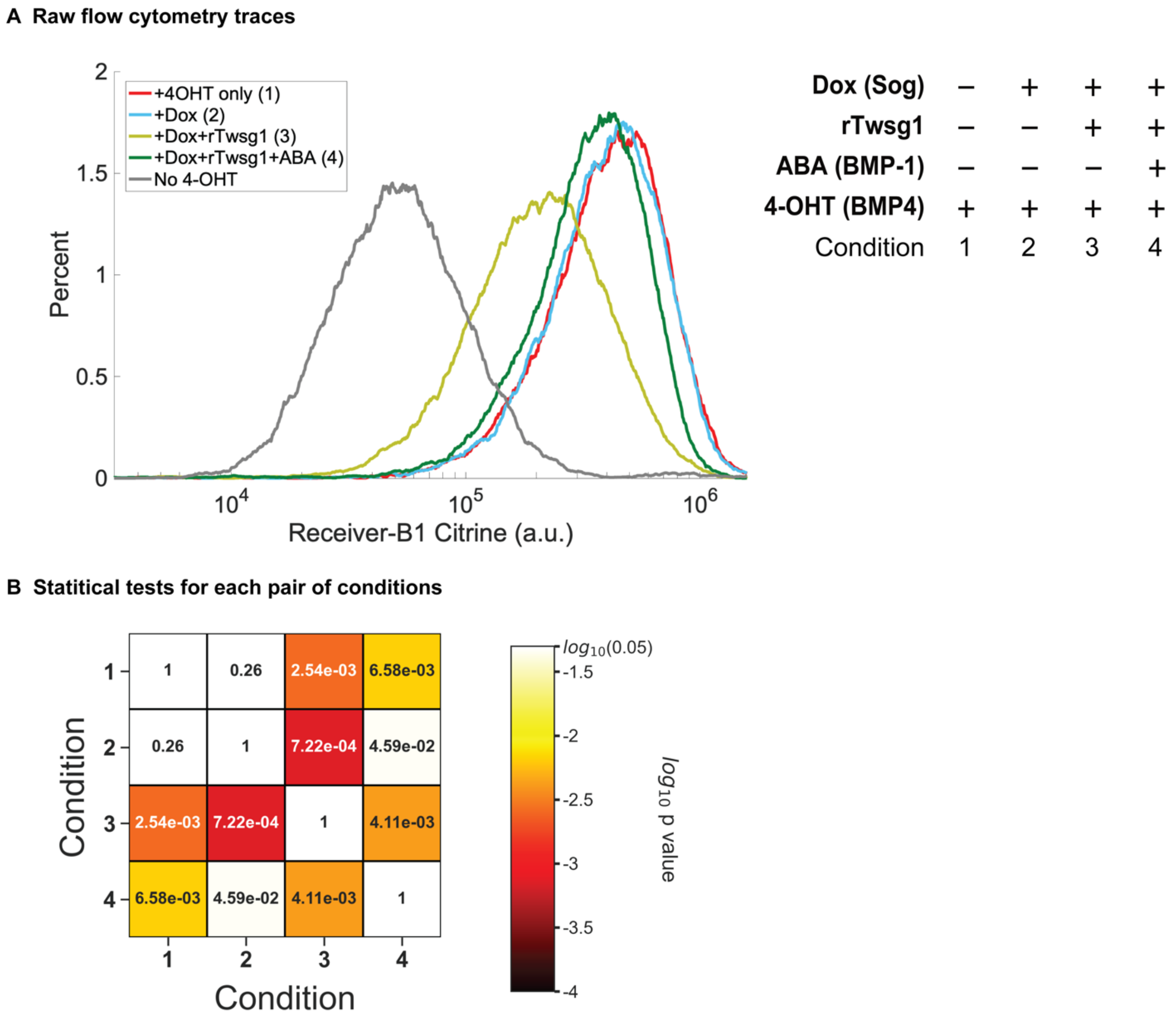

Fig. S10. Raw flow cytometry traces and statistical tests of sender-receiver co-culture experiment in Fig. 6A.

(**A**) These raw flow cytometry traces are from one of the four replicate experiments. To calculate the log2 fold change in Fig. 6A, we first obtained the mean Citrine from each trace, then calculated the log2 fold change by the equation $\log_2[(\text{Receiver Citrine} - Cit_0) / (Cit_1 - Cit_0)]$, where Cit_0 is the receiver Citrine of the sample without 4-OHT (gray trace) and Cit_1 is the receiver Citrine of the sample with only 4-OHT induction (red trace). The number in the parenthesis in the figure legend corresponds to the condition number on the right. (**B**) For each matrix entry, the number is the p value of Welch's t-test between the corresponding pair of conditions. The condition number corresponds to the condition number in (A).

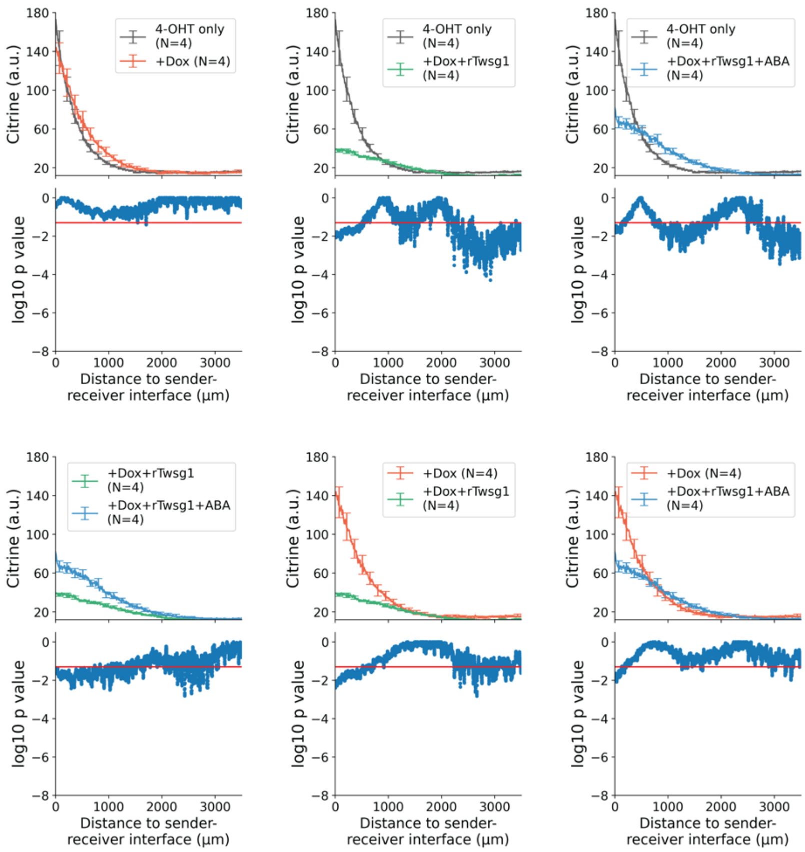

Fig. S11. Pairwise comparison of Citrine traces in Fig. 6C.
For each pair of traces, we performed pixel-wise Welch's t-test along the distance to the sender-receiver interface. The red line on the distance versus log10 p value plot is $\log_{10}(0.05)$.

Table S1. List of BMP ligands, receptors and modulators expressed in NMuMG cells (data from Antebi et al., 2017).

**Gene (Ligand)**	**FPKM**	**Gene (Receptor)**	**FPKM**	**Gene (Modulator)**	**FPKM**
Bmp2	0.966488	Bmpr1a	18.6297	Nog	0.0946048
Bmp3	0	Bmpr1b	0	Chrd	0.132197
Bmp3b	0	Bmpr2	5.64062	Twsg1	57.4595
Bmp4	6.17382	Acvr1	65.3538	Bmp1	24.9904
Bmp5	0	Acvr2a	54.2251	Bmper	0.486271
Bmp6	0.128872	Acvr2b	105.359	Bambi	0.956501
Bmp7	1.06013			Dragon/Rgmb	6.41953
Bmp8a	0.240426			Chrdl1	0
Bmp8b	0.0148391			Chrdl2	0.0308965
Bmp9	0			Fst	3.12281
Bmp10	0			Fstl1	1.01323
Bmp15	0			Fstl5	0
				Sost	0
				Sostdc-1	0
				Nbl1	37.5115
				Cer1	0
				Grem1	0
				Grem2	0
				Crim1	37.1051
				Kcp	0.433142
				Gapdh	39.5971

Table S2. List of plasmids used in this study.

Index	Construct name	Cell line
pJM009	PB-PGK-ERT2-Gal4-T2A-H2B-Citrine-SV40-HygroR	Sender-B
pJM018	PB-UAS-BMP4-IRES-H2B-mCherry-BGHpA-SV40-BlastR	Sender-B
pRZ007	PB-EF1α-TET3G-IRES-H2B-citrine-BGHpA-SV40-NeoR	Sender-C, Sender-N, Sender-S
pRZ011	PB-TRE3G-Chordin-IRES-H2B-mTurquoise2-SV40-ZeoR	Sender-C
pRZ012	PB-TRE3G-Noggin-IRES-H2B-mTurquoise2-SV40-ZeoR	Sender-N
pRZ018	PB-TRE3G-Sog-IRES-H2B-mTurquoise2-SV40-ZeoR	Sender-S
pRZ044	PB-UAS-BMP-1-IRES-H2B-mCherry-SV40-BlastR	Receiver-B1
pRZ056	PB-EF1α-NLS-VP16-PYL-IRES-NLS-Gal4DBD-ABI-SV40-NeoR	Receiver-B1
pRZ032	PB-EF1α-IRES-H2B-mTurquoise2-BGHpA-SV40-NeoR	Sender-B*
ES006	PB-CAG-H2B-mTurqoise2-BGHpA-SV40-HygroR	Sender-C*, Sender-N*, Sender-S*

Note:
PB = PiggyBac backbone;
ERT2-Gal4 = Gal4-VP16 transcriptional activator fused with human estrogen receptor (variant ERT2) (*46*);
Gal4DBD = DNA-binding domain of Gal4;
Tet3G = Tet-On 3G transactivator protein from Takara Bio;
PYL, ABI = domains from PYL1 and ABI1 genes that confer ABA-induced proximity (*47*);
PGK = constitutive promoter from mouse phosphoglycerate kinase 1 gene;
UAS = inducible promoter with ERT2-Gal4 binding site;
EF1α = constitutive EF1α promoter;
TRE3G = inducible promoter with Tet3G binding sites;
CAG = constitutive CAG promoter (*75*);
SV40 = constitutive promoter from the early promoter of the simian virus 40
NLS = nuclear localization sequence;
IRES = internal ribosome entry site;
BGHpA = bovine growth hormone polyadenylation signal;
HygroR, BlastR, NeoR, ZeoR = antibiotics resistance genes for hygromycin, blasticidin, geneticin/neomycin and zeocin, respectively;
Construct maps in GenBank format are available at data.caltech.edu/records/0sdrn-73r13.

Table S3. List of stable cell lines constructed for this study and their use in the figures.

Cell lines	Parental cells	Polyclonal or Monoclonal	Integrated constructs	Figures
Sender-B	NMuMG (ATCC)	Monoclonal	pJM009, pJM018	2-5, 6C, S2, S4C, S4D, S5, S8, Movie S1, Movie S5-8
Sender-C	NMuMG (ATCC)	Monoclonal	pRZ007, pRZ011	3B, 4, 5, S5, S8, Movie S1, Movie S5-8
Sender-N	NMuMG (ATCC)	Monoclonal	pRZ007, pRZ012	S4C, S4D
Sender-S	NMuMG (ATCC)	Monoclonal	pRZ007, pRZ018	6C
Receiver-B1	NMuMG Sensor Line from (*45*)	Monoclonal	pRZ044, pRZ056	2- 6, S1-5, S8, S10, Movie S1-8
Sender-B*	Sender-B	Monoclonal	pRZ032	3A, 6B, S3, S4B, S10
Sender-C*	Sender-C	Monoclonal	ES006	3A, S3
Sender-N*	Sender-N	Polyclonal	ES006	S4B
Sender-S*	Sender-S	Polyclonal	ES006	6B, S10

Table S4. List of parameters used in the model.

Parameters	Estimated range in the literature	Value used in the model	References
D_B, D_C, D_T, D_{BC}, D_{BCT}	0.1 – 20 μm^2/s	15 μm^2/s	(*33*, *34*, *76*, *77*)
k_R	0.00168 – 0.0438 nM^{-1}min^{-1}	0.00326 nM^{-1}min^{-1} (fitted within literature range)	(*78*–*81*)
r_R	0.018 – 0.09 min^{-1}	0.09 min^{-1} (fitted within literature range)	(*78*–*81*)
$RTotal$	0.0375 – 2.7 nM (corresponding to 18 – 1300 molecules/μm^2 and a 800 μm Matrigel layer on the cell surface)	0.57 nM (fitted within literature range)	(*82*, *83*)
γ	0.00693 – 0.173 min^{-1} (corresponding to $t_{1/2}$ of 4 – 100 min)	0.00693 min^{-1} (fitted within literature range)	(*84*–*92*)
k_{BC}	0.0168 – 0.0234x10^{-2} nM^{-1}min^{-1}	0.018 nM^{-1}min^{-1}	(*58*, *93*)
r_{BC}	0.003 – 0.204 min^{-1}	0.06 min^{-1}	(*58*, *93*)
k_{BCT}	N/A	0.018 nM^{-1}min^{-1} *	N/A
r_{BCT}	0.0554 – 0.9 min^{-1} (corresponding to Chordin-Twsg1 dissociation constant ranging 3.08 – 50 nM, calculated by chosen kBCT)	0.0554 min^{-1}	(*40*, *71*)
β	N/A	0.002 (basal) – 0.01 (fully induced) min^{-1} #	N/A
b	N/A	15.8 a.u./min (fitted)	N/A
n	N/A	1.4 (fitted)	N/A
K	N/A	0.0126 nM (fitted)	N/A
δ_{cit}	0.000481 min^{-1} (corresponding to $t_{1/2}$ of 24 hr)	0.000481 min^{-1}	(*70*)
r_{cit}	N/A	0.00385 min^{-1} (corresponding to $t_{1/2}$ of 3 hr)	Fig. S1.

* Lacking direct measurements of k_{BCT}, we set it arbitrarily to the same value as k_{BC}, which lies within the diffusion limited range. We also verified that major conclusions are not sensitive to its precise value (Fig. S6).

# Twsg has been shown to enhance Chordin cleavage by BMP-1 (*18*, *74*), but we did not find a good estimate of the magnitude of this effect. We set the cleavage rate to be the same for Chordin with or without Twsg, but also verified that major conclusions are not sensitive to whether cleavage rates are set to be the same or not (Fig. S6).

Movie S1. One replicate of the time-lapse movie of BMP4 gradients in the no induction condition.

Movie S2. One replicate of the time-lapse movie of Receiver-B1 cells turning on Citrine fluorescence after the addition of 5 ng/ml rBMP4.

Movie S3. One replicate of the time-lapse movie of Receiver-B1 cells turning on Citrine fluorescence after the addition of 10 ng/ml rBMP4.

Movie S4. One replicate of the time-lapse movie of Receiver-B1 cells turning on Citrine fluorescence after the addition of 20 ng/ml rBMP4.

Movie S5. One replicate of the time-lapse movie of BMP4 gradients in the 4 μM 4-OHT only condition.

Movie S6. Another replicate of the time-lapse movie of BMP4 gradients in the 4 μM 4-OHT only condition.

Movie S7. One replicate of the time-lapse movie of BMP4 gradients in the 4 μM 4-OHT + 30 ng/ml Dox condition.

Movie S8. One replicate of the time-lapse movie of BMP4 gradients in the 4 μM 4-OHT + 30 ng/ml Dox + 10 nM rTwsg1 + 500 μM ABA condition.

REFERENCES AND NOTES

1. G. Struhl, K. Struhl, P. M. Macdonald, The gradient morphogen *bicoid* is a concentration-dependent transcriptional activator. *Cell* **57**, 1259–1273 (1989).
2. W. Driever, C. Nüsslein-Volhard, A gradient of *bicoid* protein in *Drosophila* embryos. *Cell* **54**, 83–93 (1988).
3. W. Driever, C. Nüsslein-Volhard, The *bicoid* protein determines position in the *Drosophila* embryo in a concentration-dependent manner. *Cell* **54**, 95–104 (1988).
4. A. M. Turing, The chemical basis of morphogenesis. *Philos. Trans. R. Soc. Lond., B, Biol. Sci.* **237**, 37–72 (1952).
5. K. A. Wharton, R. P. Ray, W. M. Gelbart, An activity gradient of decapentaplegic is necessary for the specification of dorsal pattern elements in the *Drosophila* embryo. *Development* **117**, 807–822 (1993).
6. L. Dale, F. C. Wardle, A gradient of BMP activity specifies dorsal–ventral fates in early *Xenopus* embryos. *Semin. Cell Dev. Biol.* **10**, 319–326 (1999).
7. L. Marchant, C. Linker, P. Ruiz, N. Guerrero, R. Mayor, The inductive properties of mesoderm suggest that the neural crest cells are specified by a BMP gradient. *Dev. Biol.* **198**, 319–329 (1998).
8. J. Raspopovic, L. Marcon, L. Russo, J. Sharpe, Digit patterning is controlled by a Bmp-Sox9-Wnt Turing network modulated by morphogen gradients. *Science* **345**, 566–570 (2014).
9. H. Spemann, *Embryonic Development and Induction* (Taylor & Francis, 1988).
10. D. Ben-Zvi, B.-Z. Shilo, A. Fainsod, N. Barkai, Scaling of the BMP activation gradient in *Xenopus* embryos. *Nature* **453**, 1205–1211 (2008).
11. H. Inomata, T. Shibata, T. Haraguchi, Y. Sasai, Scaling of dorsal-ventral patterning by embryo size-dependent degradation of spemann's organizer signals. *Cell* **153**, 1296–1311 (2013).
12. C. E. Peluso, D. Umulis, Y.-J. Kim, M. B. O'Connor, M. Serpe, Shaping BMP morphogen gradients through enzyme-substrate interactions. *Dev. Cell* **21**, 375–383 (2011).
13. D. Umulis, M. B. O'Connor, S. S. Blair, The extracellular regulation of bone morphogenetic protein signaling. *Development* **136**, 3715–3728 (2009).
14. E. Bier, E. M. De Robertis, EMBRYO DEVELOPMENT. BMP gradients: A paradigm for morphogen-mediated developmental patterning. *Science* **348**, aaa5838 (2015).
15. S. Piccolo, Y. Sasai, B. Lu, E. M. De Robertis, Dorsoventral patterning in *Xenopus*: Inhibition of ventral signals by direct binding of chordin to BMP-4. *Cell* **86**, 589–598 (1996).
16. J. J. Ross, O. Shimmi, P. Vilmos, A. Petryk, H. Kim, K. Gaudenz, S. Hermanson, S. C. Ekker, M. B.

O'Connor, J. L. Marsh, Twisted gastrulation is a conserved extracellular BMP antagonist. *Nature* **410**, 479–483 (2001).

17. C. Chang, D. A. Holtzman, S. Chau, T. Chickering, E. A. Woolf, L. M. Holmgren, J. Bodorova, D. P. Gearing, W. E. Holmes, A. H. Brivanlou, Twisted gastrulation can function as a BMP antagonist. *Nature* **410**, 483–487 (2001).
18. I. C. Scott, I. L. Blitz, W. N. Pappano, S. A. Maas, K. W. Cho, D. S. Greenspan, Homologues of twisted gastrulation are extracellular cofactors in antagonism of BMP signalling. *Nature* **410**, 475–478 (2001).
19. O. Shimmi, M. B. O'Connor, Physical properties of Tld, Sog, Tsg and Dpp protein interactions are predicted to help create a sharp boundary in Bmp signals during dorsoventral patterning of the *Drosophila* embryo. *Development* **130**, 4673–4682 (2003).
20. A. Eldar, R. Dorfman, D. Weiss, H. Ashe, B.-Z. Shilo, N. Barkai, Robustness of the BMP morphogen gradient in *Drosophila* embryonic patterning. *Nature* **419**, 304–308 (2002).
21. B.-Z. Shilo, M. Haskel-Ittah, D. Ben-Zvi, E. D. Schejter, N. Barkai, Creating gradients by morphogen shuttling. *Trends Genet.* **29**, 339–347 (2013).
22. M. B. O'Connor, D. Umulis, H. G. Othmer, S. S. Blair, Shaping BMP morphogen gradients in the *Drosophila* embryo and pupal wing. *Development* **133**, 183–193 (2006).
23. I. C. Scott, I. L. Blitz, W. N. Pappano, Y. Imamura, T. G. Clark, B. M. Steiglitz, C. L. Thomas, S. A. Maas, K. Takahara, K. W. Cho, D. S. Greenspan, Mammalian BMP-1/Tolloid-related metalloproteinases, including novel family member mammalian Tolloid-like 2, have differential enzymatic activities and distributions of expression relevant to patterning and skeletogenesis. *Dev. Biol.* **213**, 283–300 (1999).
24. M. Serpe, D. Umulis, A. Ralston, J. Chen, D. J. Olson, A. Avanesov, H. Othmer, M. B. O'Connor, S. S. Blair, The BMP-binding protein Crossveinless 2 is a short-range, concentration-dependent, biphasic modulator of BMP signaling in *Drosophila*. *Dev. Cell* **14**, 940–953 (2008).
25. A. Ralston, S. S. Blair, Long-range Dpp signaling is regulated to restrict BMP signaling to a crossvein competent zone. *Dev. Biol.* **280**, 187–200 (2005).
26. L. Zakin, C. A. Metzinger, E. Y. Chang, C. Coffinier, E. M. De Robertis, Development of the vertebral morphogenetic field in the mouse: Interactions between Crossveinless-2 and Twisted Gastrulation. *Dev. Biol.* **323**, 6–18 (2008).
27. L. Zakin, E. Y. Chang, J.-L. Plouhinec, E. M. De Robertis, Crossveinless-2 is required for the

relocalization of Chordin protein within the vertebral field in mouse embryos. *Dev. Biol.* **347**, 204–215 (2010).

28. B. Reversade, E. M. De Robertis, Regulation of ADMP and BMP2/4/7 at opposite embryonic poles generates a self-regulating morphogenetic field. *Cell* **123**, 1147–1160 (2005).
29. S. Matsuda, O. Shimmi, Directional transport and active retention of Dpp/BMP create wing vein patterns in Drosophila. *Dev. Biol.* **366**, 153–162 (2012).
30. C. M. Mizutani, Q. Nie, F. Y. M. Wan, Y.-T. Zhang, P. Vilmos, R. Sousa-Neves, E. Bier, J. L. Marsh, A. D. Lander, Formation of the BMP activity gradient in the *Drosophila* embryo. *Dev. Cell* **8**, 915–924 (2005).
31. O. Shimmi, D. Umulis, H. Othmer, M. B. O'Connor, Facilitated transport of a Dpp/Scw heterodimer by Sog/Tsg leads to robust patterning of the *Drosophila* blastoderm embryo. *Cell* **121**, 493 (2005).
32. Y.-C. Wang, E. L. Ferguson, Spatial bistability of Dpp–receptor interactions during *Drosophila* dorsal–ventral patterning. *Nature* **434**, 229–234 (2005).
33. J. Zinski, Y. Bu, X. Wang, W. Dou, D. Umulis, M. C. Mullins, Systems biology derived source-sink mechanism of BMP gradient formation. *eLife* **6**, e22199 (2017).
34. A. P. Pomreinke, G. H. Soh, K. W. Rogers, J. K. Bergmann, A. J. Bläßle, P. Müller, Dynamics of BMP signaling and distribution during zebrafish dorsal-ventral patterning. *eLife* **6**, e25861 (2017).
35. M. Oelgeschläger, J. Larraín, D. Geissert, E. M. De Robertis, The evolutionarily conserved BMP-binding protein Twisted gastrulation promotes BMP signalling. *Nature* **405**, 757–763 (2000).
36. S. Piccolo, E. Agius, B. Lu, S. Goodman, L. Dale, E. M. De Robertis, Cleavage of Chordin by Xolloid metalloprotease suggests a role for proteolytic processing in the regulation of Spemann organizer activity. *Cell* **91**, 407–416 (1997).
37. J. Larrain, D. Bachiller, B. Lu, E. Agius, S. Piccolo, E. M. De Robertis, BMP-binding modules in chordin: A model for signalling regulation in the extracellular space. *Development* **127**, 821–830 (2000).
38. H. Troilo, A. L. Barrett, A. V. Zuk, M. P. Lockhart-Cairns, A. P. Wohl, C. P. Bayley, R. Dajani, R. B. Tunnicliffe, L. Green, T. A. Jowitt, G. Sengle, C. Baldock, Structural characterization of twisted gastrulation provides insights into opposing functions on the BMP signalling pathway. *Matrix Biol.* **55**, 49–62 (2016).
39. P. Li, J. S. Markson, S. Wang, S. Chen, V. Vachharajani, M. B. Elowitz, Morphogen gradient reconstitution reveals Hedgehog pathway design principles. *Science* **360**, 543–548 (2018).
40. R. Sekine, T. Shibata, M. Ebisuya, Synthetic mammalian pattern formation driven by differential

diffusivity of Nodal and Lefty. *Nat. Commun.* **9**, 5456 (2018).

41. H. Yano, H. Uchida, T. Iwasaki, M. Mukai, H. Akedo, K. Nakamura, S. Hashimoto, H. Sabe, Paxillin α and Crk-associated substrate exert opposing effects on cell migration and contact inhibition of growth through tyrosine phosphorylation. *Proc. Natl. Acad. Sci.* **97**, 9076–9081 (2000).
42. Y. E. Antebi, J. M. Linton, H. Klumpe, B. Bintu, M. Gong, C. Su, R. McCardell, M. B. Elowitz, Combinatorial signal perception in the BMP pathway. *Cell* **170**, 1184–1196.e24 (2017).
43. H. E. Klumpe, M. A. Langley, J. M. Linton, C. J. Su, Y. E. Antebi, M. B. Elowitz, The context-dependent, combinatorial logic of BMP signaling. *Cell Syst.* **13**, 388–407.e10 (2022).
44. S. S. Gerety, M. A. Breau, N. Sasai, Q. Xu, J. Briscoe, D. G. Wilkinson, An inducible transgene expression system for zebrafish and chick. *Development* **140**, 2235–2243 (2013).
45. F.-S. Liang, W. Q. Ho, G. R. Crabtree, Engineering the ABA plant stress pathway for regulation of induced proximity. *Sci. Signal.* **4**, rs2 (2011).
46. A. D. Lander, Q. Nie, F. Y. M. Wan, Do morphogen gradients arise by diffusion? *Dev. Cell* **2**, 785–796 (2002).
47. M. Paulsen, S. Legewie, R. Eils, E. Karaulanov, C. Niehrs, Negative feedback in the bone morphogenetic protein 4 (BMP4) synexpression group governs its dynamic signaling range and canalizes development. *Proc. Natl. Acad. Sci. U.S.A.* **108**, 10202–10207 (2011).
48. L. Zakin, E. M. De Robertis, Extracellular regulation of BMP signaling. *Curr. Biol.* **20**, R89–R92 (2010).
49. P. Tiwari, H. Rengarajan, T. E. Saunders, Scaling of internal organs during *Drosophila* embryonic development. *Biophys. J.* **120**, 4264–4276 (2021).
50. R. Mateus, L. Holtzer, C. Seum, Z. Hadjivasiliou, M. Dubois, F. Jülicher, M. Gonzalez-Gaitan, BMP signaling gradient scaling in the zebrafish pectoral fin. *Cell Rep.* **30**, 4292–4302.e7 (2020).
51. J.-L. Plouhinec, L. Zakin, Y. Moriyama, E. M. De Robertis, Chordin forms a self-organizing morphogen gradient in the extracellular space between ectoderm and mesoderm in the *Xenopus* embryo. *Proc. Natl. Acad. Sci.* **110**, 20372–20379 (2013).
52. T. Kurata, J. Nakabayashi, T. S. Yamamoto, M. Mochii, N. Ueno, Visualization of endogenous BMP signaling during *Xenopus* development. *Differentiation* **67**, 33–40 (2001).
53. J. Tucker, K. Mintzer, M. Mullins, The BMP signaling gradient patterns dorsoventral tissues in a temporally progressive manner along the anteroposterior axis. *Dev. Cell* **14**, 108–119 (2008).
54. M.-C. Ramel, C. S. Hill, The ventral to dorsal BMP activity gradient in the early zebrafish embryo is

determined by graded expression of BMP ligands. *Dev. Biol.* **378**, 170–182 (2013).

55. H. Troilo, A. V. Zuk, R. B. Tunnicliffe, A. P. Wohl, R. Berry, R. F. Collins, T. A. Jowitt, G. Sengle, C. Baldock, Nanoscale structure of the BMP antagonist chordin supports cooperative BMP binding. *Proc. Natl. Acad. Sci. U.S.A.* **111**, 13063–13068 (2014).
56. P. Blader, S. Rastegar, N. Fischer, U. Strähle, Cleavage of the BMP-4 antagonist chordin by zebrafish tolloid. *Science* **278**, 1937–1940 (1997).
57. R. W. Padgett, J. M. Wozney, W. M. Gelbart, Human BMP sequences can confer normal dorsal-ventral patterning in the *Drosophila* embryo. *Proc. Natl. Acad. Sci. U.S.A.* **90**, 2905–2909 (1993).
58. T. K. Sampath, K. E. Rashka, J. S. Doctor, R. F. Tucker, F. M. Hoffmann, *Drosophila* transforming growth factor beta superfamily proteins induce endochondral bone formation in mammals. *Proc. Natl. Acad. Sci. U.S.A.* **90**, 6004–6008 (1993).
59. G. Marqués, M. Musacchio, M. J. Shimell, K. Wünnenberg-Stapleton, K. W. Cho, M. B. O’Connor, Production of a DPP activity gradient in the early *Drosophila* embryo through the opposing actions of the SOG and TLD proteins. *Cell* **91**, 417–426 (1997).
60. A. Madamanchi, M. C. Mullins, D. M. Umulis, Diversity and robustness of bone morphogenetic protein pattern formation. *Development* **148**, dev192344 (2021).
61. H. L. Ashe, M. Levine, Local inhibition and long-range enhancement of Dpp signal transduction by Sog. *Nature* **398**, 427–431 (1999).
62. L. Wolpert, Positional information and the spatial pattern of cellular differentiation. *J. Theor. Biol.* **25**, 1–47 (1969).
63. K. Miyazono, Y. Kamiya, M. Morikawa, Bone morphogenetic protein receptors and signal transduction. *J. Biochem.* **147**, 35–51 (2009).
64. A. Bandyopadhyay, K. Tsuji, K. Cox, B. D. Harfe, V. Rosen, C. J. Tabin, Genetic analysis of the roles of BMP2, BMP4, and BMP7 in limb patterning and skeletogenesis. *PLOS Genet.* **2**, e216 (2006).
65. K.-S. Choi, C. Lee, D. M. Maatouk, B. D. Harfe, Bmp2, Bmp4 and Bmp7 are co-required in the mouse AER for normal digit patterning but not limb outgrowth. *PLOS ONE* **7**, e37826 (2012).
66. D. Ben-Zvi, A. Fainsod, B.-Z. Shilo, N. Barkai, Scaling of dorsal-ventral patterning in the *Xenopus laevis* embryo. *Bioessays* **36**, 151–156 (2014).
67. N. Kitsera, A. Khobta, B. Epe, Destabilized green fluorescent protein detects rapid removal of transcription blocks after genotoxic exposure. *Biotechniques* **43**, 222–227 (2007).
68. J.-L. Zhang, Y. Huang, L.-Y. Qiu, J. Nickel, W. Sebald, Von Willebrand factor type C domain-

containing proteins regulate bone morphogenetic protein signaling through different recognition mechanisms. *J. Biol. Chem.* **282**, 20002–20014 (2007).

69. A. Hartung, K. Bitton-Worms, M. M. Rechtman, V. Wenzel, J. H. Boergermann, S. Hassel, Y. I. Henis, P. Knaus, Different routes of bone morphogenic protein (BMP) receptor endocytosis influence BMP signaling. *Mol. Cell. Biol.* **26**, 7791–7805 (2006).
70. M. Schlosshauer, D. Baker, Realistic protein-protein association rates from a simple diffusional model neglecting long-range interactions, free energy barriers, and landscape ruggedness. *Protein Sci.* **13**, 1660–1669 (2004).
71. J. Larraín, M. Oelgeschläger, N. I. Ketpura, B. Reversade, L. Zakin, E. M. De Robertis, Proteolytic cleavage of Chordin as a switch for the dual activities of Twisted gastrulation in BMP signaling. *Development* **128**, 4439–4447 (2001).
72. J. Miyazaki, S. Takaki, K. Araki, F. Tashiro, A. Tominaga, K. Takatsu, K. Yamamura, Expression vector system based on the chicken β-actin promoter directs efficient production of interleukin-5. *Gene* **79**, 269–277 (1989).
73. S. Zhou, W.-C. Lo, J. L. Suhalim, M. A. Digman, E. Gratton, Q. Nie, A. D. Lander, Free extracellular diffusion creates the Dpp morphogen gradient of the *Drosophila* wing disc. *Curr. Biol.* **22**, 668–675 (2012).
74. A. Kicheva, P. Pantazis, T. Bollenbach, Y. Kalaidzidis, T. Bittig, F. Jülicher, M. González-Gaitán, Kinetics of morphogen gradient formation. *Science* **315**, 521–525 (2007).
75. T. Kirsch, J. Nickel, W. Sebald, BMP-2 antagonists emerge from alterations in the low-affinity binding epitope for receptor BMPR-II. *EMBO J.* **19**, 3314–3324 (2000).
76. S. Saremba, J. Nickel, A. Seher, A. Kotzsch, W. Sebald, T. D. Mueller, Type I receptor binding of bone morphogenetic protein 6 is dependent on N-glycosylation of the ligand. *FEBS J.* **275**, 172–183 (2008).
77. T. Hatta, H. Konishi, E. Katoh, T. Natsume, N. Ueno, Y. Kobayashi, T. Yamazaki, Identification of the ligand-binding site of the BMP type IA receptor for BMP-4. *Biopolymers* **55**, 399–406 (2000).
78. V. Khodr, P. Machillot, E. Migliorini, J.-B. Reiser, C. Picart, High-throughput measurements of bone morphogenetic protein/bone morphogenetic protein receptor interactions using biolayer interferometry. *Biointerphases* **16**, 031001 (2021).
79. Y. Chen, A. C. Munteanu, Y.-F. Huang, J. Phillips, Z. Zhu, M. Mavros, W. Tan, Mapping receptor density on live cells by using fluorescence correlation spectroscopy. *Chemistry* **15**, 5327–5336 (2009).

80. A. F. Eckert, P. Gao, J. Wesslowski, X. Wang, J. Rath, K. Nienhaus, G. Davidson, G. U. Nienhaus, Measuring ligand-cell surface receptor affinities with axial line-scanning fluorescence correlation spectroscopy. *eLife* **9**, e55286 (2020).
81. A. L. Schwartz, S. E. Fridovich, H. F. Lodish, Kinetics of internalization and recycling of the asialoglycoprotein receptor in a hepatoma cell line. *J. Biol. Chem.* **257**, 4230–4237 (1982).
82. A. Ciechanover, A. L. Schwartz, A. Dautry-Varsat, H. F. Lodish, Kinetics of internalization and recycling of transferrin and the transferrin receptor in a human hepatoma cell line. Effect of lysosomotropic agents. *J. Biol. Chem.* **258**, 9681–9689 (1983).
83. A. L. Schwartz, A. Bolognesi, S. E. Fridovich, Recycling of the asialoglycoprotein receptor and the effect of lysosomotropic amines in hepatoma cells. *J. Cell Biol.* **98**, 732–738 (1984).
84. G. J. Strous, A. Du Maine, J. E. Zijderhand-Bleekemolen, J. W. Slot, A. L. Schwartz, Effect of lysosomotropic amines on the secretory pathway and on the recycling of the asialoglycoprotein receptor in human hepatoma cells. *J. Cell Biol.* **101**, 531–539 (1985).
85. W. M. Pardridge, A. J. Van Herle, R. T. Naruse, G. Fierer, A. Costin, In vivo quantification of receptor-mediated uptake of asialoglycoproteins by rat liver. *J. Biol. Chem.* **258**, 990–994 (1983).
86. P. R. Dragsten, D. B. Mitchell, G. Covert, T. Baker, Drug delivery using vesicles targeted to the hepatic asialoglycoprotein receptor. *Biochim. Biophys. Acta.* **926**, 270–279 (1987).
87. C. T. H. Jonker, C. Deo, P. J. Zager, A. N. Tkachuk, A. M. Weinstein, E. Rodriguez-Boulan, L. D. Lavis, R. Schreiner, Accurate measurement of fast endocytic recycling kinetics in real time. *J. Cell Sci.* **133**, jcs231225 (2019).
88. S. N. Roed, P. Wismann, C. R. Underwood, N. Kulahin, H. Iversen, K. A. Cappelen, L. Schäffer, J. Lehtonen, J. Hecksher-Soerensen, A. Secher, J. M. Mathiesen, H. Bräuner-Osborne, J. L. Whistler, S. M. Knudsen, M. Waldhoer, Real-time trafficking and signaling of the glucagon-like peptide-1 receptor. *Mol. Cell. Endocrinol.* **382**, 938–949 (2014).
89. K. M. Mayle, A. M. Le, D. T. Kamei, The intracellular trafficking pathway of transferrin. *Biochim. Biophys. Acta.* **1820**, 264–281 (2012).
90. F. Rentzsch, J. Zhang, C. Kramer, W. Sebald, M. Hammerschmidt, Crossveinless 2 is an essential positive feedback regulator of Bmp signaling during zebrafish gastrulation. *Development* **133**, 801–811 (2006).
